# mTORC1 activity regulates post-translational modifications of glycine decarboxylase to modulate glycine metabolism and tumorigenesis

**DOI:** 10.1038/s41467-021-24321-3

**Published:** 2021-07-09

**Authors:** Rui Liu, Lin-Wen Zeng, Rong Gong, Fanen Yuan, Hong-Bing Shu, Shu Li

**Affiliations:** 1 Department of Infectious Diseases, Zhongnan Hospital of Wuhan University, Frontier Science Center for Immunology and Metabolism, Medical Research Institute, Wuhan University, Research Unit of Innate Immune and Inflammatory Diseases of the Chinese Academy of Medical Sciences, Wuhan, China; 2 Key Laboratory of Combinatorial Biosynthesis and Drug Discovery, Ministry of Education, and School of Pharmaceutical Sciences, Wuhan University, Wuhan, China; 3 Department of Neurosurgery, Renmin Hospital of Wuhan University, Wuhan University, Wuhan, China

**Keywords:** Acetyltransferases, Cancer metabolism

## Abstract

Glycine decarboxylase (GLDC) is a key enzyme of glycine cleavage system that converts glycine into one-carbon units. GLDC is commonly up-regulated and plays important roles in many human cancers. Whether and how GLDC is regulated by post-translational modifications is unknown. Here we report that mechanistic target of rapamycin complex 1 (mTORC1) signal inhibits GLDC acetylation at lysine (K) 514 by inducing transcription of the deacetylase sirtuin 3 (SIRT3). Upon inhibition of mTORC1, the acetyltransferase acetyl-CoA acetyltransferase 1 (ACAT1) catalyzes GLDC K514 acetylation. This acetylation of GLDC impairs its enzymatic activity. In addition, this acetylation of GLDC primes for its K33-linked polyubiquitination at K544 by the ubiquitin ligase NF-X1, leading to its degradation by the proteasomal pathway. Finally, we find that GLDC K514 acetylation inhibits glycine catabolism, pyrimidines synthesis and glioma tumorigenesis. Our finding reveals critical roles of post-translational modifications of GLDC in regulation of its enzymatic activity, glycine metabolism and tumorigenesis, and provides potential targets for therapeutics of cancers such as glioma.

## Abstract (web summary)

An increase in glycine decarboxylase (GLDC) activity, a key enzyme for glycine catabolism, has been associated to tumourigenesis. Here, the authors show that mTORC1 activation induces GLDC deacetylation which impairs its ubiquitin-associated degradation leading to increased GLDC activity and tumourigenesis.

## Introduction

Mechanistic target of rapamycin (mTOR) is a conserved serine–threonine kinase in the phosphoinositide-3 kinase-related kinase family, which integrates a wide array of extracellular and intracellular signals to regulate cell growth, metabolism, translation, and autophagy^1–3^. mTOR is the catalytic subunit of two distinct protein complexes, known as mTOR Complex 1 (mTORC1) and mTOR Complex 2 (mTORC2). The rapamycin-FKBP12 complex directly inhibits mTORC1, whereas mTORC2 is insensitive to acute rapamycin treatment^1^. mTORC1 is defined by its three core components: mTOR, regulatory protein associated with mTOR (RPTOR), and mammalian lethal with Sec13 protein 8 (mLST8)^4^. Dysregulation of mTORC1 activity induces highly active cell metabolism and proliferation states, which is commonly observed in many human cancers, including glioblastoma (GBM)^5,6^.

Glycine is a nonessential amino acid, which is an important residue of many proteins. Glycine can also be cleaved to yield one-carbon units, which are used for nucleotide synthesis via the tetrahydrofolate (THF) cycle^5,7^. In addition, high levels of glycine are toxic by its conversion to metabolites, such as aminoacetone and methylglyoxal^8^. The glycine cleavage system controls glycine catabolism through multi-step reactions, generating CO_2_, NH_3_, NADH, and 5,10-methylene-THF^9,10^. The glycine cleavage system is a multi-enzyme complex, consisting of glycine decarboxylase (GLDC, also called P protein), amino-methyltransferase (T protein), dihydrolipoamide dehydrogenase (L protein), and the hydrogen carrier protein (H protein)^11^. Various studies have demonstrated that glycine metabolism is essential for tumorigenesis^7,8,12^.

GLDC is a mitochondrial pyridoxal 5’-phosphate (PLP)-dependent enzyme that catalyzes the first and rate-limiting step in glycine catabolism^11^. GLDC binds glycine through its PLP cofactor to form an external aldimine that loses the carboxyl group as CO_2_ and donates the remaining aminomethylene moiety to the oxidized lipoamide arm of H protein^9^. Mutations in *GLDC* gene cause glycine accumulation, leading to neural tube defect and glycine encephalopathy (also known as nonketotic hyperglycinemia)^13,14^. It has also been demonstrated that GLDC is hyperactive in different types of cancer cells and plays a fundamental role in tumor growth. For example, increased expression of GLDC in non-small cell lung cancer-initiating cells is essential for tumorigenesis by promoting pyrimidine biosynthesis, glycolysis, and sarcosine production^15^. GLDC expression is markedly increased in MYCN-amplified neuroblastomas, which is required for neuroblastoma cell proliferation and tumorigenicity^16^.

In this study, we found that GLDC is acetylated at K514 by ACAT1 following mTORC1 inhibition. GLDC K514 acetylation inhibited its enzymatic activity, promoted its K33-linked polyubiquitination at K544 by NF-X1 and proteasomal degradation, and suppressed glioma tumor growth. Our findings suggest that GLDC activity is regulated by sequential posttranslational modifications, including acetylation and polyubiquitination, and reveal critical regulatory mechanisms of glycine metabolism and tumorigenesis.

## Results

### Inhibition of mTORC1 suppresses GLDC activity by promoting its acetylation at K514

**Fig. 1:**
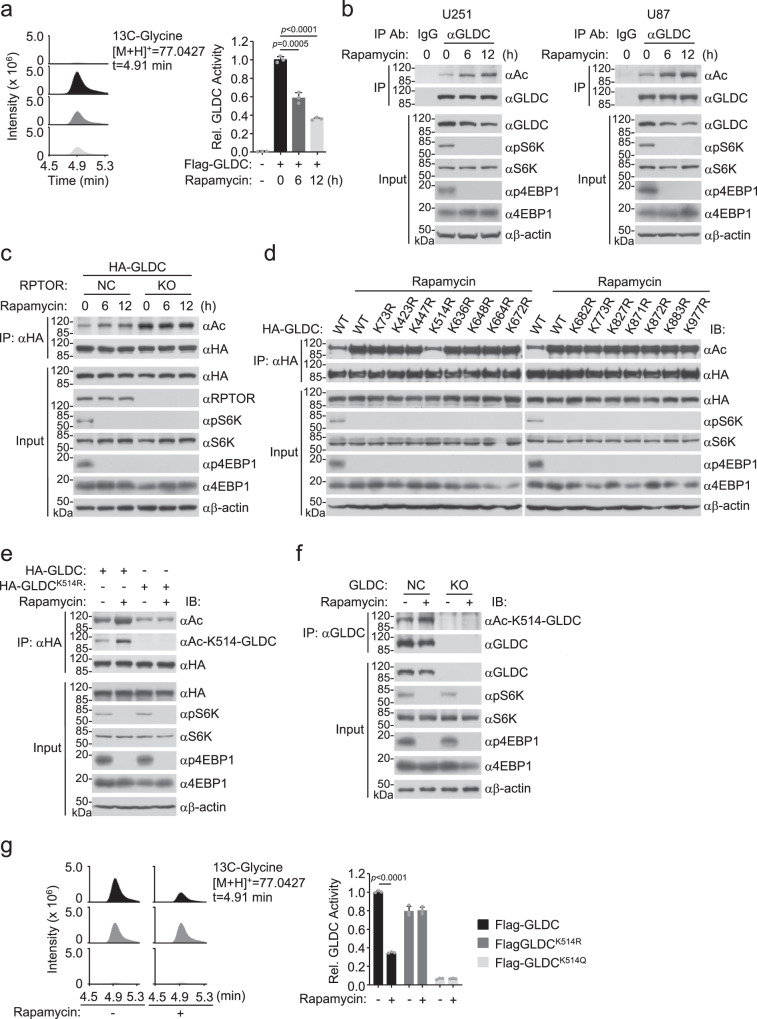
Inhibition of mTORC1 suppresses GLDC activity by promoting its acetylation at K514. **a** Rapamycin inhibits GLDC activity. U251 cells stably expressing Flag-GLDC were treated with Rapamycin (50 nM) for the indicated times. Flag-GLDC protein was purified by anti-Flag beads; equal amounts of each purified proteins (2.5 μg) were used for GLDC activity assay. Graph shows mean ± SEM, *n* = 3 technical repeats. Data were analyzed using two-way ANOVA with GraphPad Prism 7. **b** Rapamycin promotes endogenous GLDC acetylation. U251 or U87 cells were treated with DMSO or Rapamycin (50 nM) for the indicated times before co-immunoprecipitation and immunoblotting analysis with the indicated antibodies. **c** Effects of RPTOR deficiency on GLDC acetylation. Control or RPTOR-deficient HEK293 cells were transfected with the indicated plasmids for 12 h and then treated with DMSO or Rapamycin (50 nM) for the indicated times before co-immunoprecipitation and immunoblotting analysis with the indicated antibodies. **d** Effects of Rapamycin on acetylation of wild-type GLDC and its mutants. HEK293 cells were transfected with the indicated plasmids for 12 h and then treated with DMSO or Rapamycin (50 nM) for 12 h before co-immunoprecipitation and immunoblotting analysis with the indicated antibodies. **e**, **f** Rapamycin promotes GLDC K514 acetylation. HEK293 cells were transfected with the indicated plasmids for 12 h and then treated with DMSO or Rapamycin (50 nM) for 12 h before co-immunoprecipitation and immunoblotting analysis with the indicated antibodies (**e**). The control or GLDC-deficient U251 cells were treated with DMSO or Rapamycin (50 nM) for 12 h before co-immunoprecipitation and immunoblotting analysis with the indicated antibodies (**f**). **g** Effects of GLDC mutations on its activity. HEK293 cells were transfected with the indicated plasmids for 24 h and treated with DMSO or Rapamycin (50 nM) for 12 h. The Flag-tagged GLDC and its mutants were purified by anti-Flag beads and equal amounts of each purified proteins (2.5 μg) were used for GLDC activity assay. Graph shows mean ± SEM, *n* = 3 technical repeats. Data were analyzed using two-way ANOVA with GraphPad Prism 7. Source data are provided as a Source data file.

It has been shown that mTORC1 regulates certain amino acid metabolism and tumorigenesis. However, whether it regulates GLDC-mediated glycine metabolism is unknown. We generated U251 glioma cells stably expressing Flag-tagged GLDC and treated the cells with the mTORC1 inhibitor Rapamycin or left them untreated. We then purified Flag-GLDC by anti-Flag immunoaffinity beads and measured its enzymatic activity. The results indicated that Rapamycin treatment suppressed GLDC enzymatic activity (Fig. 1a). We next explored whether GLDC activity is regulated by posttranslational modifications. We found that Rapamycin treatment inhibited phosphorylation of S6K and 4EBP1 (hallmarks of mTORC1 activation) but did not affect serine/threonine or tyrosine phosphorylation of GLDC (Supplementary Fig. 1a). Interestingly, immunoblotting analysis indicated that Rapamycin treatment increased GLDC acetylation (Fig. 1b). RPTOR is a core component of mTORC1. In RPTOR-deficient cells, the basal acetylation of GLDC was increased and Rapamycin treatment did not further increase its acetylation (Fig. 1c). These results suggest that mTORC1 signal inhibits GLDC acetylation.

Analysis of proteomic databases indicates that 15 lysine residues of GLDC are potentially acetylated (https://www.phosphosite.org/). To test whether these residues are primary acetylation sites regulated by mTORC1, we generated Arg (R) (to mimic deacetylated lysine) substitution mutants of each lysine residue. We found that Rapamycin treatment induced increase of acetylation of wild-type GLDC and its 14 mutants but not the GLDC^K514R^ mutant, which had basal acetylation similar as wild-type GLDC without Rapamycin treatment (Fig. 1d). These results suggest that, while GLDC is basally acetylated at other lysines, Rapamycin induces acetylation of GLDC at K514. Human GLDC K514 is conserved across species (Supplementary Fig. 1b). Previous studies have demonstrated that K514 is localized in the catalytic pocket of GLDC^9,17^. We generated an antibody specifically recognizing K514-acetylated GLDC (anti-Ac-K514-GLDC; Supplementary Fig. 1c). Immunoblotting analysis with this antibody confirmed that GLDC K514 was acetylated and this was increased following Rapamycin treatment (Fig. 1e, f). These results suggest that K514 is the major acetylation site negatively regulated by mTORC1. To investigate the effects of K514 acetylation of GLDC, we generated GLDC^K514Q^ mutant, which mimics its K514 acetylation. We transfected Flag-tagged wild-type GLDC, GLDC^K514R^, and GLDC^K514Q^ into HEK293 cells and purified these proteins by anti-Flag immunoaffinity beads. We found that the enzymatic activity of wild-type GLDC was inhibited in Rapamycin-treated cells. GLDC^K514R^ had slightly reduced activity in comparison to wild-type GLDC, but its activity was not inhibited following rapamycin treatment. However, the enzymatic activity of GLDC^K514Q^ was impaired in untreated and Rapamycin-treated cells (Fig. 1g). Taken together, these results suggest that inhibition of mTORC1 signal suppresses GLDC enzymatic activity by promoting its K514 acetylation.

### ACAT1 mediates GLDC K514 acetylation

**Fig. 2:**
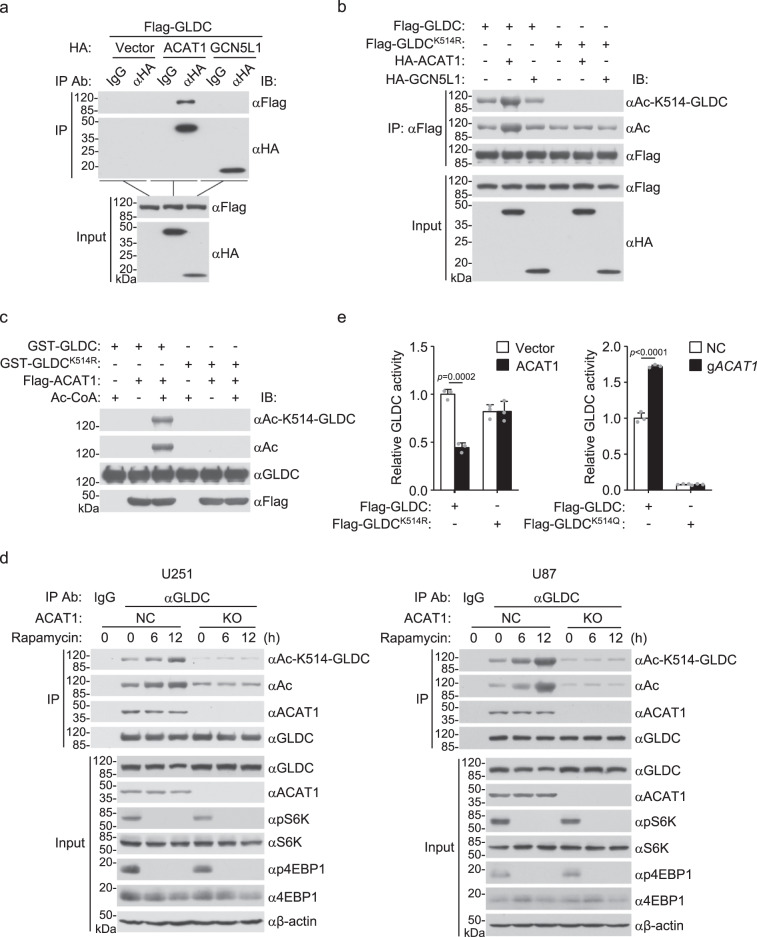
ACAT1 mediates GLDC K514 acetylation. **a** ACAT1 is associated with GLDC. HEK293 cells were transfected with the indicated plasmids for 24 h before co-immunoprecipitation and immunoblotting analysis with the indicated antibodies. **b** ACAT1 mediates GLDC acetylation at K514. HEK293 cells were transfected with the indicated plasmids for 24 h before co-immunoprecipitation and immunoblotting analysis with the indicated antibodies. **c** Effects of ACAT1 on acetylation of wild-type GLDC or GLDC^K514R^ in vitro. GST-GLDC was purified from *E. coli*. Flag-ACAT1 was purified from HEK293 cells. The purified proteins were incubated for acetylation assays, followed by immunoblotting analysis with the indicated antibodies. **d** ACAT1 deficiency inhibits Rapamycin-induced GLDC acetylation. The control or ACAT1-deficient U251 or U87 cells were treated with DMSO or Rapamycin (50 nM) for the indicated times before co-immunoprecipitation and immunoblotting analysis with the indicated antibodies. **e** Effects of ACAT1 on GLDC activity. HEK293 (left histograph) or ACAT1-deficient HEK293 (right histograph) cells were transfected with the indicated plasmids for 24 h before purification of Flag-GLDC, Flag-GLDC^K514R^, and Flag-GLDC^K514Q^. Equal amounts of each purified proteins (2.5 μg) were used for GLDC activity assay. Graph shows mean ± SEM, *n* = 3 technical repeats. Data were analyzed using two-way ANOVA with GraphPad Prism 7. Source data are provided as a Source data file.

We next investigate the enzymes that catalyze GLDC K514 acetylation. Previously, two mitochondrial acetyltransferases, including acetyl-CoA acetyltransferase 1 (ACAT1) and general control nonrepressed 5-like 1 (GCN5L1), have been identified^18,19^. Co-immunoprecipitation experiments indicated that ACAT1 but not GCN5L1 was associated with GLDC (Fig. 2a). Overexpression of ACAT1 but not GCN5L1 markedly increased acetylation of wild-type GLDC but not GLDC^K514R^ (Fig. 2b). In vitro acetylation assays further showed that ACAT1 catalyzed acetylation of wild-type GLDC, but not GLDC^K514R^ (Fig. 2c). Endogenous GLDC was constitutively associated with ACAT1, and knockout of ACAT1 by the CRISPR-Cas9 system impaired basal and Rapamycin-induced GLDC K514 acetylation in U251 or U87 cells (Fig. 2d). Consistently, overexpression of ACAT1 inhibited enzymatic activity of wild-type GLDC but not GLDC^K514R^, whereas knockout of ACAT1 increased the enzymatic activity of wild-type GLDC but not GLDC^K514Q^ (Fig. 2e). These results suggest that the acetyltransferase ACAT1 catalyzes GLDC K514 acetylation and inhibits its enzymatic activity.

### SIRT3 deacetylases GLDC at K514

**Fig. 3:**
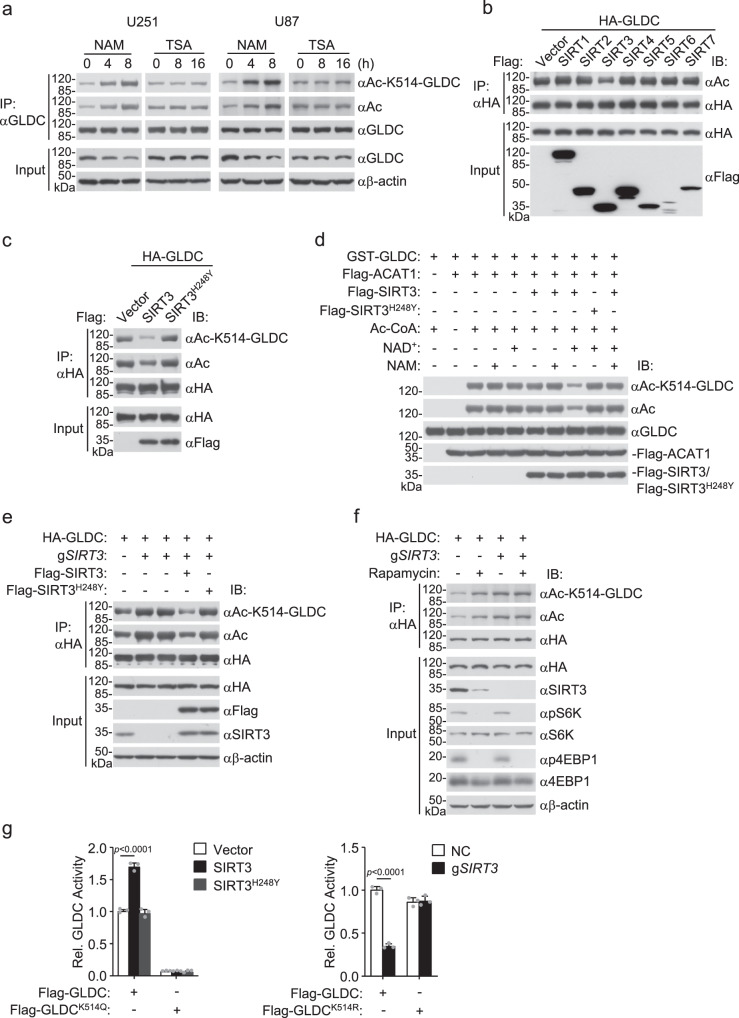
SIRT3 deacetylases GLDC at K514. **a** NAM promotes GLDC K514 acetylation. U251 or U87 cells were untreated or treated with NAM (80 μM) or TSA (100 nM) for the indicated times before co-immunoprecipitation and immunoblotting analysis with the indicated antibodies. **b** SIRT3 mediates GLDC deacetylation. HEK293 cells were transfected with the indicated plasmids for 24 h before co-immunoprecipitation and immunoblotting analysis with the indicated antibodies. **c** SIRT3 deacetylates GLDC at K514. HEK293 cells were transfected with the indicated plasmids for 24 h before co-immunoprecipitation and immunoblotting analysis with the indicated antibodies. **d** SIRT3 mediates GLDC K514 deacetylation in vitro. The indicated recombinant proteins were incubated for deacetylation assays before immunoblotting analysis with the indicated antibodies. **e** SIRT3 deficiency promotes GLDC K514 acetylation. The control or SIRT3-deficient HEK293 cells were transfected with the indicated plasmids for 24 h before co-immunoprecipitation and immunoblotting analysis with the indicated antibodies. **f** Rapamycin fails to further increase GLDC K514 acetylation in SIRT3-deficient cells. The control or SIRT3-deficient HEK293 cells were transfected with the indicated plasmids for 12 h and then untreated or treated with DMSO or Rapamycin (50 nM) for 12 h before co-immunoprecipitation and immunoblotting analysis with the indicated antibodies. **g** Effects of SIRT3 or SIRT3^H248Y^ on GLDC activity. HEK293 (left histograph) or SIRT3-deficient HEK293 (right histograph) cells were transfected with the indicated plasmids for 24 h before purification of Flag-GLDC, Flag-GLDC^K514Q^, and Flag-GLDC^K514R^. Equal amounts of each purified proteins (2.5 μg) were used for GLDC activity assay. Graph shows mean ± SEM, *n* = 3 technical repeats. Data were analyzed using two-way ANOVA with GraphPad Prism 7. Source data are provided as a Source data file.

Lysine deacetylation is mediated by two protein families, including the nicotinamide adenine dinucleotide (NAD^+^)-dependent deacetylases (sirtuins (SIRTs)) and the zinc-dependent histone deacetylases (HDACs)^20^. We found that treatment of U251 or U87 cells with nicotinamide (NAM, an inhibitor of SIRTs) but not Trichostatin A (TSA, an inhibitor of HDACs) markedly increased GLDC K514 acetylation (Fig. 3a). Overexpression of SIRT3 but not the other 6 SIRTs reduced GLDC pan-acetylation (Fig. 3b). The enzymatic inactive mutant of SIRT3, SIRT3^H248Y^, also lost its ability to inhibit GLDC K514 acetylation (Fig. 3c). In vitro assays confirmed that SIRT3 but not SIRT3^H248Y^ mediated GLDC K514 deacetylation (Fig. 3d). Knockout of SIRT3 increased GLDC K514 acetylation, which was reversed by complement with wild-type SIRT3 but not SIRT3^H248Y^ (Fig. 3e). Rapamycin failed to further increase GLDC K514 acetylation in SIRT3-deficient cells (Fig. 3f). Consistently, overexpression of SIRT3 but not SIRT3^H248Y^ increased wild-type GLDC but not GLDC^K514Q^ enzymatic activity, whereas knockout of SIRT3 inhibited it (Fig. 3g). These results suggest that SIRT3 mediates deacetylation of GLDC at K514 and increases its enzymatic activity.

### mTORC1 signal upregulates SIRT3

**Fig. 4:**
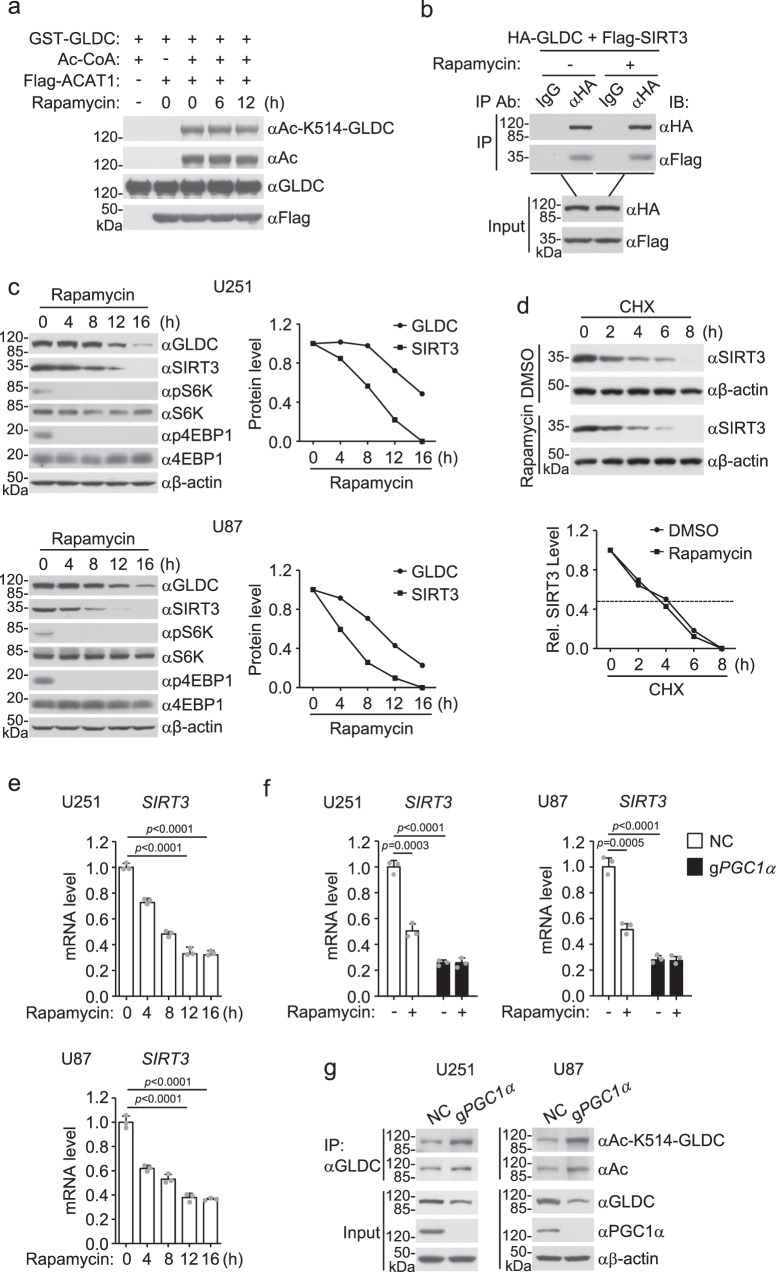
mTORC1 signal upregulates SIRT3. **a** Effects of ACAT1 on GLDC acetylation. Recombinant GST-GLDC was purified from *E. coli*. Flag-ACAT1 was immunoprecipitated from HEK293 cells after treatment with Rapamycin (50 nM) for the indicated times. The purified proteins were incubated for acetylation assay, followed by immunoblotting analysis with the indicated antibodies. **b** Effects of Rapamycin on GLDC and SIRT3 interaction. HEK293 cells were transfected with the indicated plasmids for 12 h and then treated with DMSO or Rapamycin (50 nM) for the indicated times before co-immunoprecipitation and immunoblotting analysis with the indicated antibodies. **c** Rapamycin downregulates SIRT3 levels. U251 or U87 cells were treated with DMSO or Rapamycin (50 nM) for the indicated times before immunoblotting analysis with the indicated antibodies. The GLDC or SIRT3 band intensities relative to the corresponding β-actin bands were shown in the histograph. **d** Effects of Rapamycin treatment on SIRT3 degradation. U251 cells were pretreated with DMSO or Rapamycin (50 nM) for 6 h, then treated with CHX (0.1 mM) for the indicated times before immunoblots with the indicated antibodies. The SIRT3 band intensities relative to the corresponding β-actin bands are shown in the histograph. **e** Rapamycin inhibits SIRT3 mRNA levels. U251 or U87 cells were treated with DMSO or Rapamycin (50 nM) for the indicated times before qPCR analysis of mRNA levels of the indicated genes. Graph shows mean ± SEM, *n* = 3 independent samples from one representative experiment. Data were analyzed using two-way ANOVA with GraphPad Prism 7. **f** PGC1α deficiency inhibits SIRT3 mRNA levels. Control and PGC1α-deficient U251 or U87 cells were treated with DMSO or Rapamycin (50 nM) for 6 h before qPCR analysis of mRNA levels of the indicated genes. Graph shows mean ± SEM, *n* = 3 independent samples from one representative experiment. Data were analyzed using two-way ANOVA with GraphPad Prism 7. **g** PGC1α deficiency increases acetylation of GLDC at K514. Control and PGC1α-deficient U251 or U87 cells were lysed. Co-immunoprecipitation and immunoblotting analysis were performed with the indicated antibodies. Source data are provided as a Source data file.

In our early experiments, we found that Rapamycin had no marked effects on the protein levels of ACAT1 as well as its association with GLDC (Fig. 2d). Rapamycin treatment also did not affect the ability of ACAT1 to acetylate GLDC (Fig. 4a). These results suggest that mTORC1 signal does not affect ACAT1 activity. In addition, Rapamycin also did not induce interaction between SIRT3 and GLDC (Fig. 4b). In contrast, we found that the protein levels of SIRT3 and GLDC in U251 or U87 cells were markedly downregulated following Rapamycin treatment (Fig. 4c). Treatment of U251 cells with the protein synthesis inhibitor cycloheximide (CHX) did not affect Rapamycin-induced downregulation of SIRT3 (Fig. 4d), suggesting that regulation of SIRT3 level by Rapamycin is independent on posttranslational mechanisms. Quantitative PCR (qPCR) analysis indicated that Rapamycin inhibited *SIRT3* mRNA levels in U125 or U87 cells (Fig. 4e). Previous studies suggest that peroxisome proliferator-activated receptor gamma coactivator 1-α (PGC1α) is a crucial transcription factor for *Sirt3* gene^21,22^. It has also been shown that mTORC1 activity induces transcription of PGC1α, which is mediated by the transcription factor YY1^23^. Consistently, knockout of *PGC1α* in U251 or U87 cells inhibited *SIRT3* mRNA level, which was not further inhibited by Rapamycin treatment (Fig. 4f). In addition, knockout of PGC1α increased the acetylation of GLDC at K514 and decreased its abundance (Fig. 4g). Together, these results suggest that mTORC1 signal transcriptionally induces SIRT3 levels through PGC1α.

### GLDC K514 acetylation promotes its K33-linked polyubiquitination and proteasomal degradation

**Fig. 5:**
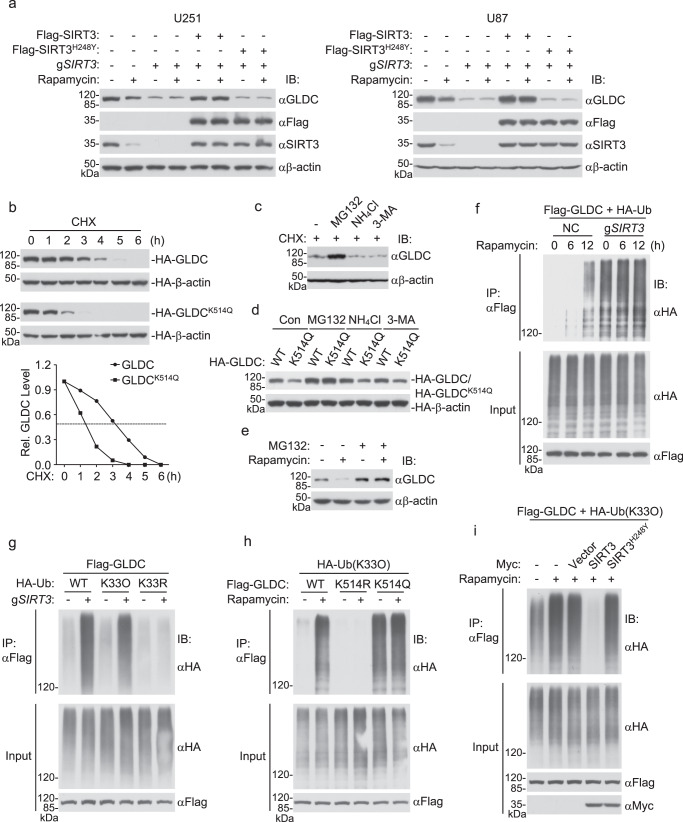
GLDC K514 acetylation promotes its K33-linked polyubiquitination and proteasomal degradation. **a** SIRT3 deficiency downregulates GLDC levels. The control and SIRT3-deficient U251 or U87 cells were reconstituted with wild-type SIRT3 or SIRT3^H248Y^ and then untreated or treated with DMSO or Rapamycin (50 nM) for 12 h before immunoblotting analysis with the indicated antibodies. **b** Half-lives of wild-type GLDC and GLDC^K514Q^. HEK293 cells transfected with wild-type GLDC or GLDC^K514Q^ were untreated or treated with CHX (0.1 mM) for the indicated times before immunoblotting analysis. The wild-type GLDC or GLDC^K514Q^ band intensities relative to the corresponding HA-β-actin bands are shown in the histograph. **c** MG132 inhibits GLDC degradation. U251 cells were treated with CHX (0.1 mM), MG132 (100 μM), NH4Cl (25 mM), or 3-MA (500 ng/ml) as indicated for 4 h before immunoblotting analysis with the indicated antibodies. **d** MG132 inhibits the degradation of GLDC^K514Q^. HEK293 cells were transfected with wild-type GLDC or GLDC^K514Q^ for 20 h. The cells were then treated with MG132 (100 μM), NH4Cl (25 mM), or 3-MA (500 ng/ml) for 6 h before immunoblotting analysis with the indicated antibodies. **e** MG132 inhibits Rapamycin-induced degradation of GLDC. U251 cells were treated with DMSO or Rapamycin (50 nM) for 10 h and then treated with MG132 (100 μM) for 6 h before immunoblotting analysis with the indicated antibodies. **f** Rapamycin promotes GLDC polyubiquitination. Control or SIRT3-deficient HEK293 cells were transfected with the indicated plasmids for 12 h and then treated with DMSO or Rapamycin (50 nM) for the indicated times before co-immunoprecipitation and immunoblotting analysis with the indicated antibodies. **g** SIRT3 deficiency promotes K33-linked polyubiquitination of GLDC. Control or SIRT3-deficient HEK293 cells were transfected with the indicated plasmids for 24 h before co-immunoprecipitation and immunoblotting analysis with the indicated antibodies. **h** Effects of K514 mutation of GLDC on its K33-linked polyubiquitination. HEK293 cells were transfected with the indicated plasmids for 12 h and then treated with DMSO or Rapamycin (50 nM) for 12 h before co-immunoprecipitation and immunoblotting analysis with the indicated antibodies. **i** Overexpression of SIRT3 abolished rapamycin-induced K33-linked polyubiquitination of GLDC. HEK293 cells were transfected with the indicated plasmids for 12 h and then treated with DMSO or Rapamycin (50 nM) for the indicated times before co-immunoprecipitation and immunoblotting analysis with the indicated antibodies. Source data are provided as a Source data file.

In our above experiments, we found that both SIRT3 and GLDC were downregulated following Rapamycin treatment of U251 or U87 cells (Fig. 4c). However, Rapamycin did not affect the mRNA levels of GLDC gene in U251 or U87 cells (Supplementary Fig. 2a). In light of these and other results, we hypothesized that GLDC K514 acetylation following SIRT3 downregulation promotes its degradation. Consistently, we found that SIRT3 deficiency caused downregulation of GLDC protein levels, which was reversed by reconstitution with wild-type SIRT3 but not SIRT3^H248Y^ in U251 or U87 cells (Fig. 5a). SIRT3 deficiency did not affect *GLDC* mRNA levels in U251 or U87 cells (Supplementary Fig. 2b). GLDC^K514Q^, a mutant that mimics K514 acetylation, had a shorter half-life than wild-type GLDC (Fig. 5b). These results suggest that GLDC K514 acetylation following SIRT3 downregulation promotes its degradation.

To further investigate the mechanisms responsible for acetylation-primed degradation of GLDC, we treated U251 cells with inhibitors for various protein degradation pathways. The proteasome inhibitor MG132, but not the lysosome inhibitor ammonium chloride (NH_4_Cl) or autophagosome inhibitor 3-methyladenine (3-MA), markedly inhibited GLDC degradation after termination of protein synthesis by CHX (Fig. 5c). Mutation of K514 to Q caused downregulation of GLDC, which was inhibited by MG132 but not NH_4_Cl or 3-MA (Fig. 5d). MG132 also inhibited Rapamycin-induced degradation of GLDC (Fig. 5e). These results suggest that GLDC K514 acetylation primes it for proteasomal degradation.

We further investigated the mechanisms leading to GLDC proteasomal degradation. We found that Rapamycin treatment as well as SIRT3 deficiency induced polyubiquitination of GLDC (Fig. 5f). Utilizing ubiquitin mutants in which one or six lysine residues are replaced with Arginine (R), we found that SIRT3 deficiency increased K33-linked polyubiquitination but not other lysine residue-linked polyubiquitination of GLDC (Fig. 5g and Supplementary Fig. 2c). Consistently, Rapamycin treatment induced K33-linked polyubiquitination of wild-type GLDC but not GLDC^K514R^, whereas GLDC^K514Q^ was constitutively modified by K33-linked polyubiquitination (Fig. 5h). These results suggest that K514 acetylation of GLDC promotes its K33-linked polyubiquitination and proteasomal degradation. Consistently, ectopic expression of SIRT3 abolished K33-linked polyubiquitination of GLDC induced by Rapamycin (Fig. 5i).

### NF-X1 mediates K33-linked polyubiquitination of GLDC

**Fig. 6:**
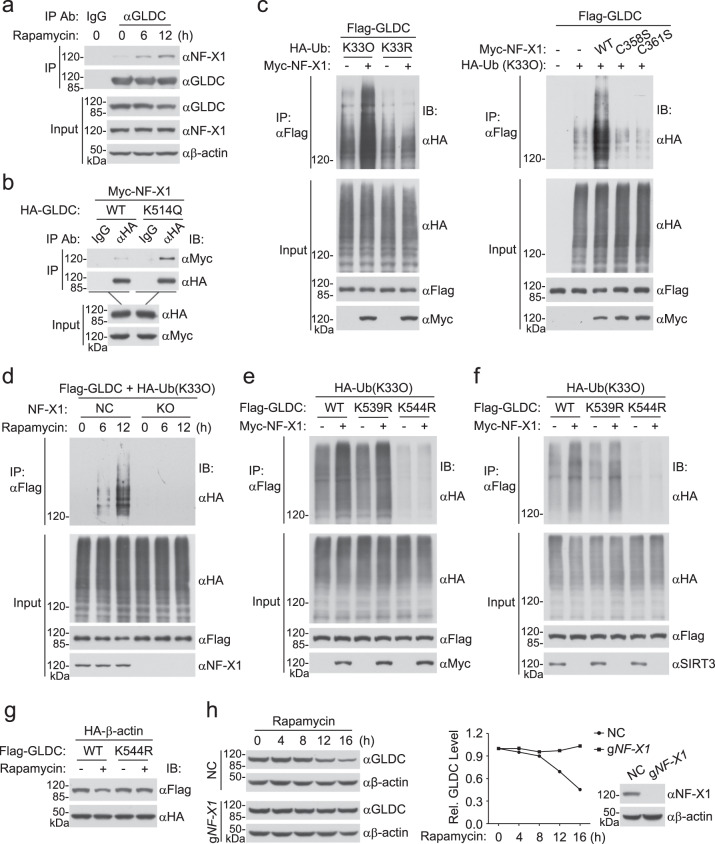
NF-X1 mediates K33-linked polyubiquitination of GLDC. **a** Association of GLDC with NF-X1. U251 cells were treated with DMSO or Rapamycin (50 nM) for the indicated times before co-immunoprecipitation and immunoblotting analysis with the indicated antibodies. **b** GLDC^K514Q^ interacts with NF-X1 better than wild-type GLDC. HEK293 cells were transfected with the indicated plasmids for 24 h before co-immunoprecipitation and immunoblotting analysis with the indicated antibodies. **c** NF-X1 promotes K33-linked polyubiquitination of GLDC. HEK293 cells were transfected with the indicated plasmids for 24 h before co-immunoprecipitation and immunoblotting analysis with the indicated antibodies. **d** NF-X1 deficiency impairs Rapamycin-induced K33-linked polyubiquitination of GLDC. Control or NF-X1-deficient HEK293 cells were transfected with the indicated plasmids for 12 h and then treated with DMSO or Rapamycin (50 nM) for the indicated times before co-immunoprecipitation and immunoblotting analysis with the indicated antibodies. **e** NF-X1 increases K33-linked polyubiquitination of wild-type GLDC and GLDC^K539R^ but not GLDC^K544R^. HEK293 cells were transfected with the indicated plasmids for 24 h before co-immunoprecipitation and immunoblotting analysis with the indicated antibodies. **f** SIRT3 deficiency increases K33-linked polyubiquitination of wild-type GLDC and GLDC^K539R^ but not GLDC^K544R^. Control or SIRT3-deficient HEK293 cells were transfected with the indicated plasmids for 24 h before co-immunoprecipitation and immunoblotting analysis with the indicated antibodies. **g** Rapamycin induces downregulation of wild-type GLDC but not GLDC^K544R^. HEK293 cells were transfected with the indicated plasmids for 12 h and then treated with DMSO or Rapamycin (50 nM) for 16 h before immunoblotting analysis with the indicated antibodies. **h** NF-X1 deficiency inhibits Rapamycin-induced degradation of GLDC. Control or NF-X1-deficient U251 cells were untreated or treated with Rapamycin (50 nM) for the indicated times before immunoblotting analysis. The GLDC band intensities relative to the corresponding β-actin bands are shown in the histograph. Source data are provided as a Source data file.

We next attempted to identify the E3 ubiquitin ligases that catalyze K33-linked polyubiquitination of GLDC. We expressed HA-tagged GLDC in HEK293 cells, and then GLDC-bound proteins were immunoprecipitated with anti-HA and analyzed by mass spectrometry (MS). Among the 279 proteins identified, 9 are E3 ubiquitin ligases (Supplementary Data 1). Overexpression experiments indicated that only NF-X1 promoted K33-linked polyubiquitination of GLDC. NF-X1 contains a RING-type zinc finger domain and has been shown to be a potential E3 ubiquitin ligase^24^. Endogenous co-immunoprecipitation experiments indicated that GLDC was basally associated with NF-X1 and Rapamycin treatment promoted their association (Fig. 6a). In addition, GLDC^K514Q^ interacted with NF-X1 better than wild-type GLDC (Fig. 6b). These results suggest that Rapamycin-induced GLDC K514 acetylation promoted its association with NF-X1. Overexpression of NF-X1 but not its inactive mutants (C358 or C361 mutated to serine) promoted K33-linked polyubiquitination of GLDC (Fig. 6c). NF-X1 deficiency impaired K33-linked polyubiquitination of GLDC induced by Rapamycin treatment (Fig. 6d). These results suggest that NF-X1 catalyzes K33-linked polyubiquitination of GLDC following its K514 acetylation induced by mTORC1 inhibition.

**Fig. 7:**
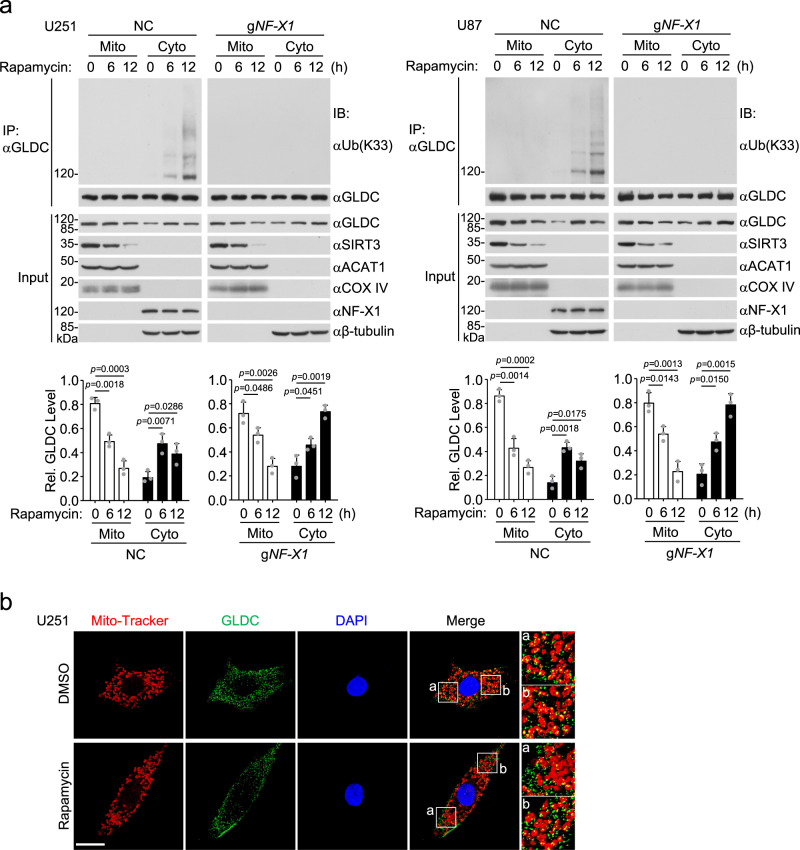
Inhibition of mTORC1 promotes GLDC cytoplasm translocation. **a** Rapamycin promotes GLDC cytoplasm translocation. Control or NF-X1-deficient U251 (left) or U87 (right) cells were untreated or treated with Rapamycin (50 nM) for the indicated times before subcellular fractionation experiments. Subcellular fractions were co-immunoprecipitated and analyzed by immunoblotting with the indicated antibodies. The band intensities of mitochondrial or cytosolic GLDC relative to the total levels are shown in the lower histographs. Mito mitochondria, Cyto cytosol. Graph shows mean ± SEM, *n* = 3 independent experiments. Data were analyzed using two-way ANOVA with GraphPad Prism 7. Source data are provided as a Source data file. **b** The subcellular localization of GLDC. U251 cells were treated with DMSO or Rapamycin (50 nM) for 12 h, then further treated with Mito-Tracker^TM^ Red CMXRos (red) for 30 min. The cells were fixed and stained with anti-GLDC (green) and DAPI (blue) before confocal microscopy. Scale bar, 20 μm.

We then attempted to identify the residues of GLDC that are conjugated with K33-linked polyubiquitin chains by NF-X1. We first examined which parts of GLDC were polyubiquitinated by NF-X1. The results indicated that NF-X1 catalyzed polyubiquitination of GLDC(489–740) but not GLDC(1–488) or GLDC(741–1020) (Supplementary Fig. 3a). We then individually mutated each of the 13 lysine residues within aa489–740 of GLDC to arginine and examined whether the mutants could be modified by K33-linked polyubiquitination. The results indicated that mutation of K544 but not the other 12 lysine residues of GLDC to arginine dramatically reduced its K33-linked polyubiquitination (Supplementary Fig. 3b). Both overexpression of NF-X1 and SIRT3 deficiency increased K33-linked polyubiquitination of wild-type GLDC and GLDC^K539R^ but not GLDC^K544R^ (Fig. 6e, f). In addition, overexpression of NF-X1 caused downregulation of wild-type GLDC and all the mutants except for GLDC^K544R^ (Supplementary Fig. 3c). Sequence analysis showed that K544 of GLDC was conserved in various vertebrate species (Supplementary Fig. 3d). These results suggest that NF-X1 targets GLDC K544 for K33-linked polyubiquitination. Consistently, Rapamycin induced downregulation of wild-type GLDC but not GLDC^K544R^ (Fig. 6g), whereas NF-X1 deficiency prevented Rapamycin-induced GLDC degradation in U251 (Fig. 6h) or U87 (Supplementary Fig. 3e) cells. Cellular fractionation experiments indicated that NF-X1 was located in the cytoplasm but not mitochondria, and Rapamycin treatment induced increase of GLDC in the cytoplasm and its decrease in the mitochondrion fraction of U251 and U87 cells (Fig. 7a). In addition, the results also showed that Rapamycin induced K33-linked polyubiquitination of GLDC in the cytoplasm but not in the mitochondria (Fig. 7a). Confocal microscopy further confirmed that Rapamycin treatment induced increase of GLDC in the cytoplasm (Fig. 7b). These results suggest that acetylated GLDC may be translocated from mitochondria to the cytoplasm for NF-X1-mediated polyubiquitination and degradation.

### GLDC K514 acetylation inhibits glycine catabolism and pyrimidine synthesis

**Fig. 8:**
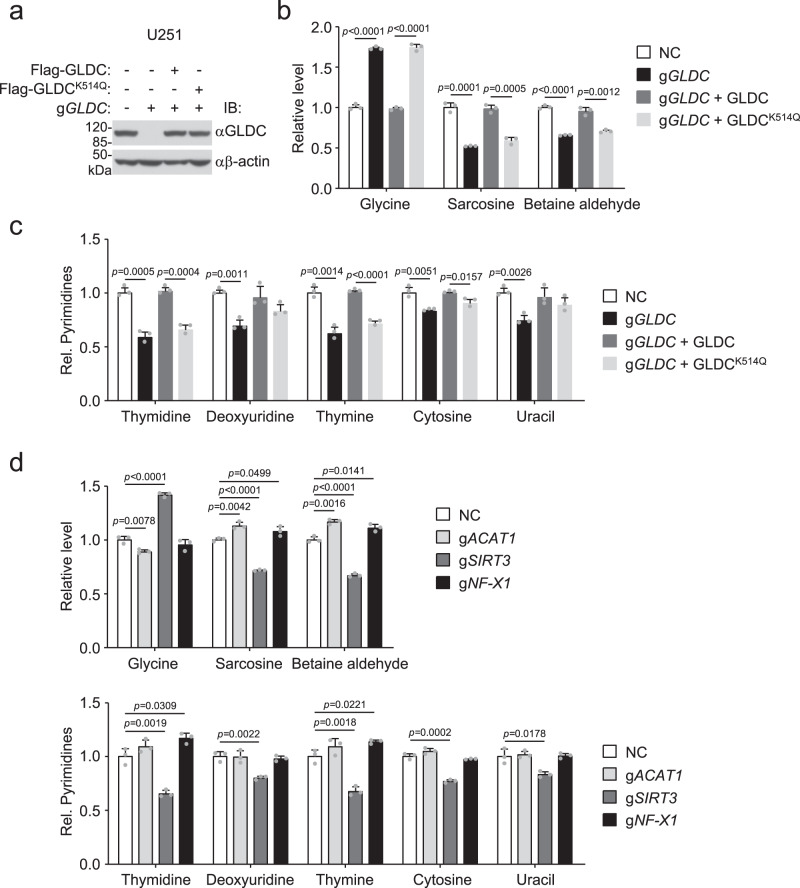
GLDC K514 acetylation inhibits glycine catabolism and pyrimidine synthesis. **a**–**c** Reconstitution of GLDC-deficient U251 cells with wild-type GLDC or GLDC^K514Q^. Lysates of the indicated cells were analyzed by immunoblots with the indicated antibodies (**a**). The control and reconstituted cells were analyzed for levels of glycine and glycine-related metabolites (**b**) and pyrimidines (**c**) by LC-HRMS. Graph shows mean ± SEM, *n* = 3 technical repeats. Data were analyzed using two-way ANOVA with GraphPad Prism 7. **d** Effects of ACAT1, SIRT3, or NF-X1 deficiency on cellular levels of glycine, glycine-related metabolites, and pyrimidines. Levels of glycine and its metabolites in the indicated cells were measured by LC-HRMS. Graph shows mean ± SEM, *n* = 3 technical repeats. Data were analyzed using two-way ANOVA with GraphPad Prism 7. Source data are provided as a Source data file.

To investigate the functional significance of GLDC K514 acetylation, we reconstituted GLDC-knockout U251 or U87 cells with Flag-tagged wild-type GLDC or GLDC^K514Q^ (Fig. 8a and Supplementary Fig. 4a). We found that GLDC deficiency increased glycine levels, and GLDC^K514Q^-reconstituted cells had increased glycine levels in comparison to wild-type GLDC-reconstituted U251 (Fig. 8b) or U87 (Supplementary Fig. 4b) cells. Consistently, the levels of glycine-related metabolites, such as sarcosine (*N*-methylglycine), betaine aldehyde, and pyrimidines, including thymidine, deoxyuridine, thymine, cytosine, and uracil, were decreased in GLDC-deficient or GLDC^K514Q^-reconstituted compared to wild-type U251(Fig. 8b, c) or U87 (Supplementary Fig. 4b, c) cells. SIRT3 deficiency promoted glycine accumulation and suppressed production of its metabolites, whereas ACAT1 deficiency and NF-X1 deficiency had the opposite effects in these cells (Fig. 8d and Supplementary Fig. 4d). The GLDC-catalyzed reaction converts glycine into CH_2_-THF, which is a substrate for thymidylate synthesis^10,15,25^. We further investigated the role of GLDC K514 acetylation in nucleotide synthesis utilizing 13C-2-glycine isotope tracers. U87 or U251 cells were cultured in 50% extracellular 13C-2-glycine (0.4 mM glycine + 0.4 mM 13C-2-glycine)-containing medium for 24 h. Tracer analysis showed that approximately a quarter of intracellular glycine was 13C-labeled and the consumed glycine was incorporated into nucleotides (Supplementary Fig. 5a, b). The levels of labeled thymidine and thymine were impaired in GLDC-deficient compared to wild-type cells (Supplementary Fig. 5b). Reconstitution of GLDC-deficient cells with wild-type GLDC but not GLDC^K514Q^ rescued nucleotide synthesis (Supplementary Fig. 5b). These results suggest that GLDC K514 acetylation inhibits glycine catabolism and pyrimidine synthesis.

### GLDC K514 acetylation suppresses tumorigenesis

**Fig. 9:**
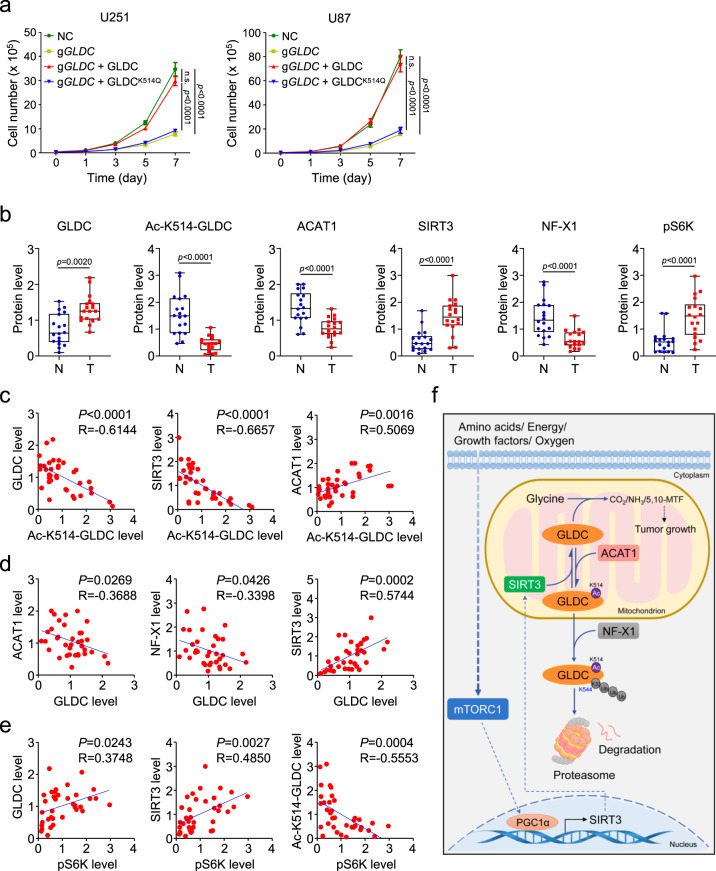
GLDC K514 acetylation suppresses tumorigenesis. **a** GLDC deficiency suppresses cell proliferation. Control and GLDC-deficient U251 or U87 cells reconstituted with wild-type GLDC or GLDC^K514Q^ were subjected to proliferation analysis. Graph shows mean ± SEM, *n* = 3 technical repeats. Data were analyzed using two-way ANOVA with GraphPad Prism 7. **b** Relative levels of the indicated proteins or their modifications. Relative levels of the indicated proteins or their modifications in Fig. S7 were plotted based on densitometry quantitation of the indicated proteins or their modifications with the ImageJ software. The GLDC, ACAT1, SIRT3, or NF-X1 band intensities relative to the corresponding β-actin bands, the Ac-K514-GLDC band intensities relative to the corresponding GLDC bands, and the p-P70 S6K band intensities relative to the corresponding P70 S6K bands are shown in the histograph. Graph shows mean ± SEM, *n* = 18 biologically independent samples. The line in the box is the median value. The bottom and top of the box are the 25th and 75th percentiles of the samples. The bottom and top of the whiskers are the minimum and maximum values of the samples. Data were analyzed using two-way ANOVA with GraphPad Prism 7. **c**–**e** Analysis of correlation of GLDC K514 acetylation with GLDC, SIRT3, and ACAT1 (**c**), GLDC with ACAT1, NF-X1, and SIRT3 (**d**), and p-p70 S6K with GLDC, SIRT3, and GLDC K514 acetylation (**e**) in GBM samples. Relative levels of the indicated proteins or their modifications are based on results from Fig. 9b and Fig. S7. Graph shows scatter plot, *n* = 36 biologically independent samples. Data were analyzed using two-tailed unpaired *t* test with GraphPad Prism 7. **f** A working model on regulation of GLDC activity by coordinated acetylation and polyubiquitination. See the text for details. Source data are provided as a Source data file.

We next investigated the biological functions of GLDC K514 acetylation. Cell proliferation assays showed that GLDC deficiency dramatically inhibited U251 or U87 cell proliferation, which was reversed by reconstitution with wild-type GLDC but not GLDC^K514Q^ (Fig. 9a). To test whether inhibition of cell proliferation by GLDC deficiency are mediated through depletion of the one-carbon pool, we replenished the one-carbon pool by adding formate. Formate contains a single carbon atom and reacts directly with THF, making formyl-THF a one-carbon donor for purine synthesis^25,26^. The results showed that formate treatment only partially rescued the proliferation of GLDC deficiency or GLDC^K514Q^ cells (Supplementary Fig. 6a). These results suggest that reduced purine biosynthesis partially contributes to the inhibition of cell proliferation caused by GLDC deficiency. In addition, tumor growth in nude mice intracranially injected with wild-type GLDC-reconstituted U251 or U87 glioma cells was markedly faster than those injected with GLDC-deficient or GLDC^K514Q^-reconstituted cells (Supplementary Fig. 6b). These results suggest that GLDC K514 acetylation suppresses cell proliferation and tumor development.

To further investigate the clinical significance of GLDC K514 acetylation, we collected 18 non-tumor (N) and 18 GBM (T) tissues from patients and performed quantitative immunoblotting analysis. The results indicated that GBM tissues had increased levels of GLDC, SIRT3, and pS6K (a hallmark of mTORC1 activity) and decreased levels of ACAT1, NF-X1, and GLDC K514 acetylation in comparison to non-tumor tissues (Fig. 9b and Supplementary Fig. 7). Furthermore, GLDC K514 acetylation levels were negatively correlated with GLDC and SIRT3 levels and positively correlated with ACAT1 levels (Fig. 9c). GLDC levels were negatively correlated with ACAT1 and NF-X1 levels but positively correlated with SIRT3 levels (Fig. 9d). Finally, mTORC1 activities were positively correlated with GLDC and SIRT3 levels but negatively correlated with levels of GLDC K514 acetylation (Fig. 9e). Taken together, these data suggest that GLDC K514 acetylation plays a crucial role in regulation of tumorigenesis of GBM.

## Discussion

Posttranslational modifications such as phosphorylation, sumoylation, acetylation, and polyubiquitination are known to be critical for regulation of protein functions^27–30^. Although the abnormal expression of GLDC has been shown to promote tumorigenesis, it is unknown whether and how GLDC is regulated by posttranslational modifications and how its posttranslational modifications contribute to cancer progression. In this study, we demonstrated that acetylation of GLDC K514 impairs its enzymatic activity. In addition, this acetylation of GLDC also primes for its K33-linked polyubiquitination and proteasomal degradation, leading to suppression of glycine metabolism and tumorigenesis. Our findings underscore the essential roles of GLDC posttranslational modifications in regulation of glycine metabolism and tumorigenesis.

mTORC1 is a central regulator of cell growth conserved from yeast to mammals, which integrates intracellular and environmental signals to regulate cell growth, autophagy, translation, and metabolism^1^. In our experiments, we found that constitutive mTORC1 activity in cells induced transcription of the deacetylase SIRT3. Overexpression of SIRT3 but not its inactive mutant SIRT3^H248Y^ deacetylated GLDC. Knockout of SIRT3 increased GLDC acetylation, which was reversed by complement with wild-type SIRT3 but not SIRT3^H248Y^. Inhibition of mTORC1 by Rapamycin increased GLDC acetylation but failed to further increase GLDC acetylation in SIRT3-deficient cells. These results suggest that mTORC1 activity in cells leads to deacetylation of GLDC by SIRT3.

Our experiments suggest that inhibition of mTORC1 activity causes GLDC acetylation at K514 by the mitochondrial acetyltransferase ACAT1. Both overexpression and in vitro acetylation experiments indicated that ACAT1 increased acetylation of wild-type GLDC but not GLDC^K514R^. Knockout of ACAT1 impaired basal and Rapamycin-induced GLDC K514 acetylation.

Several lines of evidence suggest that GLDC acetylation at K514 primes it for K33-linked polyubiquitination at K544 and proteasomal degradation. First, inhibition of mTORC1 activity by Rapamycin or SIRT3 deficiency, which increased GLDC acetylation at K514, caused GLDC degradation that was inhibited by the proteasomal inhibitor MG132. Second, the GLDC^K514Q^ mutant, which mimics K514 acetylation, had a shorter half-life than wild-type GLDC. Third, Rapamycin treatment as well as SIRT3 deficiency induced K33-linked polyubiquitination of GLDC. Rapamycin treatment induced K33-linked polyubiquitination of wild-type GLDC but not of GLDC^K514R^, whereas GLDC^K514Q^ was constitutively modified by K33-linked polyubiquitination.

Our experiments further identified NF-X1 as the E3 ubiquitin ligase responsible for K33-linked polyubiquitination of GLDC at K544. GLDC was constitutively associated with NF-X1, which was increased following Rapamycin treatment. Overexpression of NF-X1 but not its inactive mutants promoted K33-linked polyubiquitination of GLDC, whereas NF-X1 deficiency impaired Rapamycin-induced K33-linked polyubiquitination of GLDC. Our experiments also identified K544 of GLDC as the target residue for K33-linked polyubiquitination by NF-X1. Mutation of K544 of GLDC to arginine impaired its K33-linked polyubiquitination as well as NF-X1-induced degradation. Overexpression of NF-X1 and SIRT3 deficiency failed to catalyze K33-linked polyubiquitination of GLDC^K544R^. In addition, NF-X1 deficiency or mutation of K544 of GLDC to arginine prevented Rapamycin-induced GLDC degradation.

In addition to acetylation-primed polyubiquitination and proteasomal degradation, our experiments indicated that acetylation of GLDC at K514 itself impairs its enzymatic activity. In vitro experiments with purified proteins indicated that the enzymatic activity of GLDC^K514Q^, which mimics its K514 acetylation, was impaired. In addition, in vitro experiments with GLDC purified from Rapamycin-treated or SIRT3-deficient cells had reduced enzymatic activities, whereas GLDC purified from ACAT-deficient cells had increased enzymatic activities.

Based on our results, we propose a model on the regulatory mechanisms of GLDC activity. Constitutive mTORC1 activity in cancerous cells induces expression of the deacetylase SIRT3, which maintains GLDC in un-acetylated and enzymatically active state, leading to increased glycine metabolism. Inhibition of mTORC1 activity, such as in untransformed cells, causes acetylation of GLDC at K514, which is localized in its catalytic pocket by the mitochondrial acetyltransferase ACAT1. This acetylation of GLDC inactivates its enzymatic activity by physical hindrance of its substrates or causing its conformational changes. The acetylation of GLDC at K514 also promotes its mitochondrion to cytoplasm translocation and subsequent K33-linked polyubiquitination at K544 by the E3 ubiquitin ligase NF-X1 and proteasomal degradation in the cytoplasm (Fig. 9f). Consistent with this model, our experiments indicated that overexpression of SIRT3 increased GLDC enzymatic activity, whereas overexpression of ACAT1 inhibited it. In comparison to wild-type GLDC, GLDC^K514Q^ showed decreased enzymatic activity and mediated increased glycine accumulation and decreased production of its metabolites. Deficiency of SIRT3 inhibited GLDC activity and caused increased glycine accumulation and decreased production of its metabolites, whereas deficiency of ACAT1 or NX-F1 had opposite effects. GLDC deficiency dramatically inhibited glioma cell proliferation, which was reversed by reconstitution with wild-type GLDC but not GLDC^K514Q^. Tumor growth in nude mice intracranially injected with wild-type GLDC-reconstituted glioma cells was markedly faster than those injected with GLDC-deficient or GLDC^K514Q^-reconstituted cells. Analysis of human GBM tissues indicated that levels of GLDC, SIRT3, and pS6K were positively associated with tumorigenesis, whereas levels of ACAT1, NF-X1, and GLDC K514 acetylation were negatively associated with tumorigenesis. In conclusion, our findings reveal that GLDC activity is regulated by coordinated posttranslational modifications including acetylation and polyubiquitination, and this contributes to regulation of glycine metabolism and tumorigenesis. Thus, targeting of GLDC posttranslational modifications may provide a potential strategy for therapeutics of cancers.

## Methods

### Cells

U251 cells, U87 cells, and HEK293 cells were obtained from ATCC, cultured in Dulbecco’s modified Eagle’s medium (DMEM; Sigma) supplemented with 10% fetal bovine serum (FBS; Biological Industries) and 1% penicillin–streptomycin (Thermo Fisher Scientific) at 37 °C with 5% CO_2_. All cells were negative for mycoplasma.

### Animal studies

BALB/c nude mice were housed with 5 mice per cage on a 12-h light/dark cycle in a temperature-controlled room (23–25 °C) and relative humidity of 40–70% with free access to water and food. Mice were allowed at least 7 days for acclimation before experimentation. Eight-to-10-week-old and age-matched male mice were used in all experiments. All animals were randomly divided and injected intracranially with 5 × 10^5^ (in 5 μl of DMEM per mouse) U251 or U87 cells expressing the NC, g*GLDC*, g*GLDC* + Flag-GLDC, and g*GLDC* + Flag-GLDC^K514Q^. Tumor formation and phenotype were determined by histological analysis of hematoxylin and eosin-stained sections. All animal use and experimental protocols were carried out in compliance with the Institutional Animal Care and Use Committee guidelines and approved by the Animal Care and Ethics Committee of Wuhan University Medical Research Institute.

### Reagents and antibodies

Reagents and antibodies used in this study were purchased from the following indicated companies: Rapamycin (Cell Signal Technology), NAM (MedChemExpress), TSA (MedChemExpress), Lipofectamine 2000 (InvivoGen), polybrene (Millipore), SYBR (Bio-Rad), digitonin (Sigma), formate (Sigma), and 13C-2-glycine (Sigma). Information on the commercially available antibodies used in this study is provided in Supplementary Table 1. The antibody that specifically recognizes acetylated K514 of GLDC was raised by immunizing rabbits with a synthetic peptide of human GLDC (_510_GSVF(K-Ac)RTSP_518_) by ABclonal Technology (Wuhan).

### Constructs

Mammalian expression plasmids for Flag- or HA-tagged GLDC, ACAT1, GCN5L1, SIRT3, and their mutants and Myc-tagged NF-X1 and its mutants were constructed by standard molecular biology techniques. Guide RNA (gRNA) plasmids targeting GLDC, RPTOR, PGC1α, SIRT3, and NF-X1 were constructed into a lentiCRISPR V2 vector, which was provided by Dr. Shu-Wen Wu (Wuhan University).

### Recombinant protein purification

The pGEX-6p-1-GST plasmids encoding wild-type GLDC and GLDC^K514R^ (aa36–1020) were transformed into BL21-competent cells. Expression of the proteins was induced with 0.05 mM IPTG at 16 °C for 24 h. The proteins were purified with GST resins and eluted with elution buffer (phosphate-buffered saline (PBS), 100 mM Tris-HCl pH 8.8, 40 mM reduced glutathione). To prepare Flag-ACAT1, Flag-SIRT3, and Flag-SIRT3^H248Y^, mammalian expression plasmids for Flag-ACAT1, Flag-SIRT3, and Flag-SIRT3^H248Y^ were transfected into HEK293 cells. The cells were lysed 24 h after transfection. Flag antibody-conjugated beads were then used for immunoprecipitation for 4 h at 4 °C. The beads were washed three times with lysis buffer. The Flag-tagged ACAT1, SIRT3, and SIRT3^H248Y^ were eluted with 3×Flag peptides in 250 mM Tris-HCl, pH 8.0. The Flag-tagged ACAT1, SIRT3^WT^, and SIRT3^H248Y^ were used for acetylation and deacetylation assays in vitro.

### Human tissue samples

Human glioma and non-glioma tissues were collected from the Department of Neurosurgery, Renmin Hospital of Wuhan University, Wuhan, China. Non-glioma tissues were collected during surgery of severe traumatic brain injury after informed consent from the patients who needed post-trauma surgery. The clinical glioma specimens were examined and diagnosed by pathologists at Renmin Hospital of Wuhan University. Tissue procurement and use in this study were performed with written patient informed consents and approved by the Institutional Ethics Committee of Renmin Hospital of Wuhan University. The detailed clinicopathologic characteristics of patients is presented in Supplementary Table 2.

### CRISPR-Cas9 knockout

Double-stranded oligonucleotides corresponding to the target sequences were cloned into the Lenti-CRISPR-V2 vector, which were co-transfected with the packaging plasmids into HEK293 cells. Two days after transfection, the viruses were harvested, ultra-filtrated (0.45 μm filter, Millipore), and used to infect U251 cells or HEK293 cells in the presence of polybrene (8 μg/ml). The infected cells were selected with puromycin (1 μg/ml) for at least 6 days. The information of gRNA sequences is shown in Supplementary Table 3.

### Transfection

HEK293 cells were transfected by standard calcium phosphate precipitation. U251 and U87 cells were transfected with Lipofectamine 2000. The empty control plasmid was added to ensure that each transfection receives the same amount of total DNA.

### Co-immunoprecipitation, ubiquitination, and immunoblotting analysis

Cells were lysed in 1 ml of NP-40 lysis buffer (20 mM Tris-HCl, 150 mM NaCl, 1 mM EDTA, 1% Nonidet P-40, 1% Triton X-100, 10 μg/ml aprotinin, 10 μg/ml leupeptin, and 1 mM phenylmethylsulfonyl fluoride). For each immunoprecipitation reaction, a 0.4-ml aliquot of lysate was incubated with 0.5–2 μg of the indicated antibody or control IgG and 35 μl of a 1:1 slurry of Protein-G Sepharose (GE Healthcare) at 4 °C for 3 h. The Sepharose beads were washed three times with 1 ml of lysis buffer containing 500 mM NaCl. The precipitates were fractionated by sodium dodecyl sulfate–polyacrylamide gel electrophoresis (SDS-PAGE), and immunoblotting analysis was performed with the indicated antibodies. For ubiquitination assays, the immunoprecipitants were re-extracted in NP-40 lysis buffer containing 1% SDS and denatured by heating for 10 min. The supernatants were diluted with regular lysis buffer until the concentration of SDS was decreased to 0.1%, following by re-immunoprecipitation with the indicated antibodies. The immunoprecipitants were analyzed by immunoblotting with the ubiquitin antibody.

### Quantitative PCR

Total RNA was isolated for qPCR analysis to measure mRNA abundance of the indicated genes. Data shown are the relative abundance of the indicated mRNAs normalized to that of glyceraldehyde 3-phosphate dehydrogenase. The qPCR data were collected with Bio-Rad CFX96 (Version 3.1) and analyzed with Bio-Rad CFX Manager (Version 3.1). Gene-specific primer sequences are listed in Supplementary Table 4.

### Mass spectrometry

HEK293 cells (1 × 10^8^) were transfected with HA-tagged human GLDC. HA-tagged GLDC was immunoprecipitated and desalted. MS analysis was performed by SpecAlly (Wuhan) Life Science and Technology Company as previously described^31^.

### In vitro acetylation and deacetylation assay

The recombinant GST-GLDC and Flag-ACAT1 were purified and mixed in the buffer containing 40 mM Tris-HCl pH 8.0, 75 mM potassium chloride (KCl), and 10 μM acetyl CoA in a final volume of 30 μl. The reaction was incubated at 30 °C for 45 min and terminated by the addition of SDS-PAGE sample buffer and acetylated proteins were analyzed by immunoblots. For deacetylation assay, purified recombinant GST-GLDC and SIRT3 were incubated in 20 μl of deacetylation buffer (50 mM Tris–HCl pH 9.0, 4 mM MgCl_2_, 50 mM NaCl, 0.5 mM dithiothreitol (DTT), 0.5 μM TSA) with or without 10 μM Ac-CoA, 1 mM NAD^+^, and NAM at 37 °C for 3 h with gentle agitation. For NAM treatment as a control, reactions were pretreated with 10 mM NAM for 10 min. The reaction was terminated by SDS/PAGE sample buffer, and the protein samples were subjected to immunoblotting analysis with the indicated antibodies.

### Liquid chromatography–high-resolution MS (LC-HRMS)

LC-HRMS analysis was performed on a C_18_ column (Inertsil ODS-3, 4.6 by 250 mm, 5 μM) with the elution gradient of 5–25% methanol–0.15% methanoic acid over 20 min at a flow rate of 0.5 ml/min. LC-HRMS was conducted on an electrospray ionization–ion trap mass spectrometer (Thermo LTQ Obitrap Elite) in a positive mode with drying gas (275 °C, 10 l/ml) and a nebulizer pressure of 30 lb/in^2^.

### Isotope tracing

For isotope tracing studies, 1 × 10^7^ U87 or U251 cells were cultured in DMEM (0.4 mM unlabeled glycine) supplemented with 10% FBS (Biological Industries) and 0.4 mM 13C-2-glycine (Sigma). Intracellular metabolites were extracted by lysing cells in ice-cold 100% methanol. Samples were centrifuged for 20 min at 13,000 × *g*, and the supernatant was collected and analyzed by LC-HRMS.

### GLDC activity assay

Human GLDC and mutants were expressed in U251 or HEK293 cells and purified by immunoprecipitation. GLDC activity was determined by measuring the exchange of the carboxyl carbon of glycine against 13C-bicarbonate carbon^32^. The standard assay mixture, in a total volume of 300 μl at 30 °C, contains 100 mM sodium phosphate (pH 6.0), 0.1 mM PLP, 18 mM glycine, 1 mM DTT, 5 μg H protein, 2.5 μg GLDC protein, and 30 mM 13C-NaHCO_3_. All rates were corrected by control reactions without glycine. All enzyme assays were done in triplicates and repeated at least once with independent protein preparations. Immediately after starting the reactions by the addition of 13C-NaHCO_3_ for a total time of 20 min, aliquots of 200 μl were mixed with 20 μl of trichloroacetic acid. These samples were dried overnight to remove remaining 13C-NaHCO_3_; the 13C-glycine was quantified by LC-HRMS.

### Subcellular fractionation

Cells were treated with Rapamycin for various times and lysed by douncing 40 times in 1 ml of homogenization buffer (10 mmol/l Tris-HCl, pH 7.4, 2 mmol/l MgCl_2_, 10 mmol/l KCl, 250 mmol/l sucrose). The homogenized samples were centrifuged at 500 × *g* for 10 min. The supernatants were centrifuged at 5000 × *g* for 10 min to precipitate crude mitochondria, and the supernatants were saved as cytoplasm fractions^33^.

### Confocal microscopy

U251 cells were treated with dimethyl sulfoxide or Rapamycin (50 nM). Twelve hours after treatment, the cells were fixed with 4% paraformaldehyde for 15 min and permeabilized with 0.3% Triton X-100 in PBS for 15 min. The cells were blocked with 5% bovine serum albumin in PBS and stained with the indicated primary and secondary antibodies. The nuclei were strained with 4,6-diamidino-2-phenylindole for 2 min and then washed with PBS for 3 times. The stained cells were observed with a Zeiss LSM880 confocal microscope under a ×63 oil objective.

### Proliferation assay

Cells were seeded in 6-well plates at 5 × 10^4^ and allowed to adhere for 24 h. Triplicate wells were seeded for each experimental condition. Cells were trypsinized, resuspended in DMEM containing 10% FBS, and counted with a Cellometer (Bio Red) every 2 days over a 7-day period.

### Statistics and reproducibility

Data were analyzed using a Student’s unpaired *t* test, multiple *t* test, or two-way analysis of variance with GraphPad Prism 7. For the correlation study, data were analyzed using a Spearman rank correlation test. The number of asterisks represents the degree of significance with respect to *p* values, with the latter presented within each figure or figure legend. All the biochemical experiments, particularly immunoblotting analysis, were repeated for at least two times with similar results.

### Reporting summary

Further information on research design is available in the Nature Research Reporting Summary linked to this article.

## Supplementary information

Supplementary information

Description of Additional Supplementary Files

Supplementary Data 1

Reporting summary

## Data Availability

All the data supporting the findings of this study are available within the article and its supplementary information files or can be obtained from the corresponding author upon reasonable request. A reporting summary for this article is available as a Supplementary Information file. Source data are provided with this paper.

## Source data

Source Data
