# Supplementary material for: mTORC1 activity regulates post-translational modifications of glycine decarboxylase to modulate glycine metabolism and tumorigenesis
Source: Nat Commun. 2021 Jul 9;12:4227. doi: 10.1038/s41467-021-24321-3 (PMC8270999; doi:10.1038/s41467-021-24321-3)
Supplement: Supplementary file 1 — Supplementary information [file 41467_2021_24321_MOESM1_ESM.pdf]

Supplementary Information

mTORC1 activity regulates post-translational modifications of glycine decarboxylase to modulate glycine metabolism and tumorigenesis

Rui Liu[1], Lin-Wen Zeng[1], Rong Gong[2], Fanen Yuan[3], Hong-Bing Shu[1], and Shu Li[1*]

[1]Department of Infectious Diseases, Zhongnan Hospital of Wuhan University, Frontier Science Center for Immunology and Metabolism, Medical Research Institute, Wuhan University; Research Unit of Innate Immune and Inflammatory Diseases of the Chinese Academy of Medical Sciences, Wuhan 430071, China

[2]Key Laboratory of Combinatorial Biosynthesis and Drug Discovery, Ministry of Education, and School of Pharmaceutical Sciences, Wuhan University, Wuhan 430071, China

[3]Department of Neurosurgery, Renmin Hospital of Wuhan University, Wuhan University, Wuhan 430071, China

Correspondence: shuli@whu.edu.cn (S.L.)

Supplementary Figure 1-7

Supplementary Table 1-4

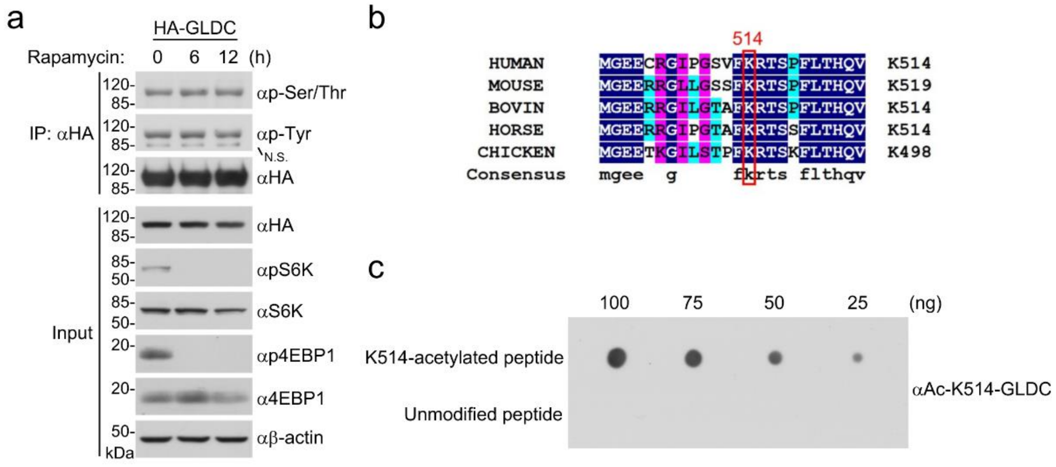

Supplementary Fig. 1. mTORC1 inhibition promotes GLDC acetylation. Related to Figure 1.

(a) Effects of Rapamycin on phosphorylation of GLDC. HEK293 cells were transfected with the indicated plasmids for 12 h and then treated with DMSO or Rapamycin (50 nM) for the indicated times before co-immunoprecipitation and immunoblotting analysis with the indicated antibodies.

(b) Sequence alignment of GLDC from the indicated species. The sequences are corresponding to aa500-524 of hGLDC.

(c) Specificity of the antibody against acetyl-K514 of GLDC. Nitrocellulose membrane was spotted with different amounts of un-modified peptides or acetylated-K514 peptides and probed with the Ac-K514-GLDC antibody.

Source data are provided as a source data file.

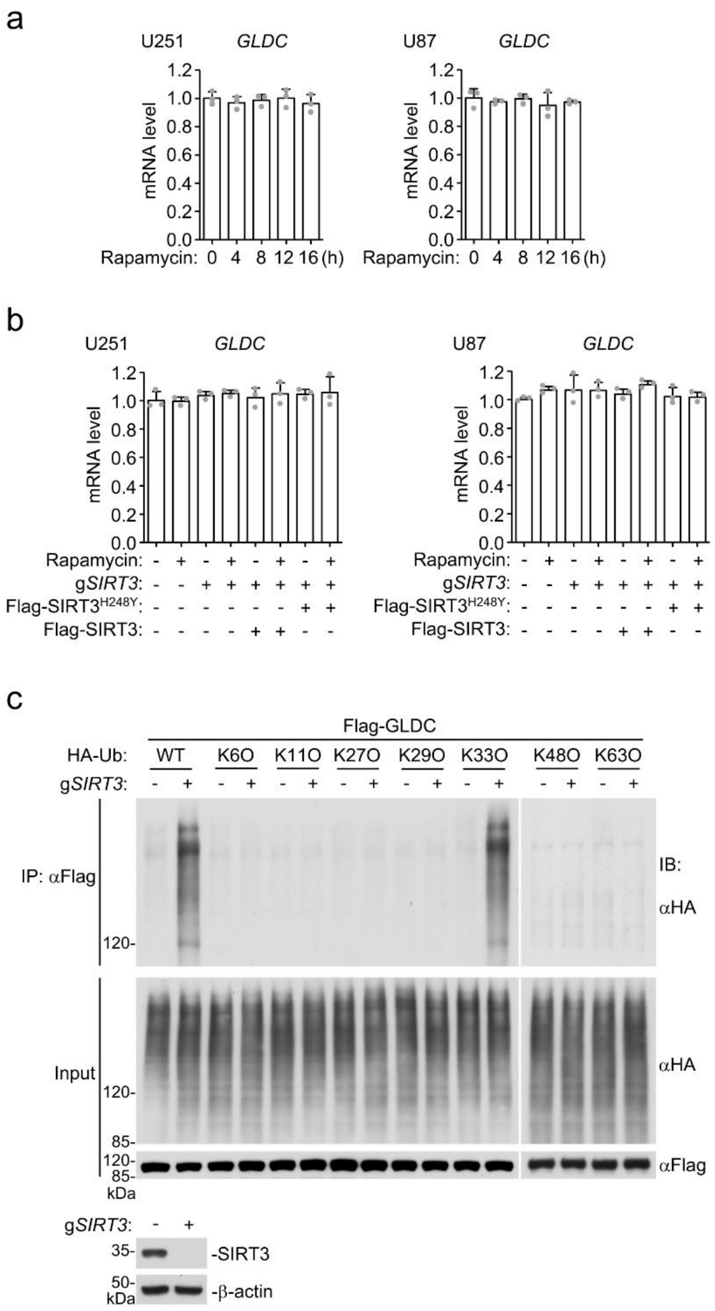

Supplementary Fig. 2. GLDC K514 acetylation promotes its K33-linked polyubiquitination and proteasomal degradation. Related to Figure 5.

(a) Effects of mTORC1 inhibition on transcription of *GLDC*. U251 or U87 cells were treated with DMSO or Rapamycin (50 nM) for the indicated times before qPCR analysis of mRNA levels of the indicated genes. Graph shows mean ± SEM, n = 3 independent samples from one representative experiment. Data were analyzed using two-way ANOVA with GraphPad Prism 7.

(b) Effects of SIRT3-deficiency on transcription of *GLDC*. The control and SIRT3-deficient U251 or U87 cells were reconstituted with wild-type SIRT3 or SIRT3^{H248Y} and then untreated or treated with DMSO or Rapamycin (50 nM) for 12 h before qPCR analysis of mRNA levels of the indicated genes. Graph shows mean ± SEM, n = 3 independent samples from one representative experiment. Data were analyzed using two-way ANOVA with GraphPad Prism 7.

(c) Effects of SIRT3-deficiency on GLDC polyubiquitination. Control or SIRT3-deficient HEK293 cells were transfected with the indicated plasmids for 24 h before co-immunoprecipitation and immunoblotting analysis with the indicated antibodies.

Source data are provided as a source data file.

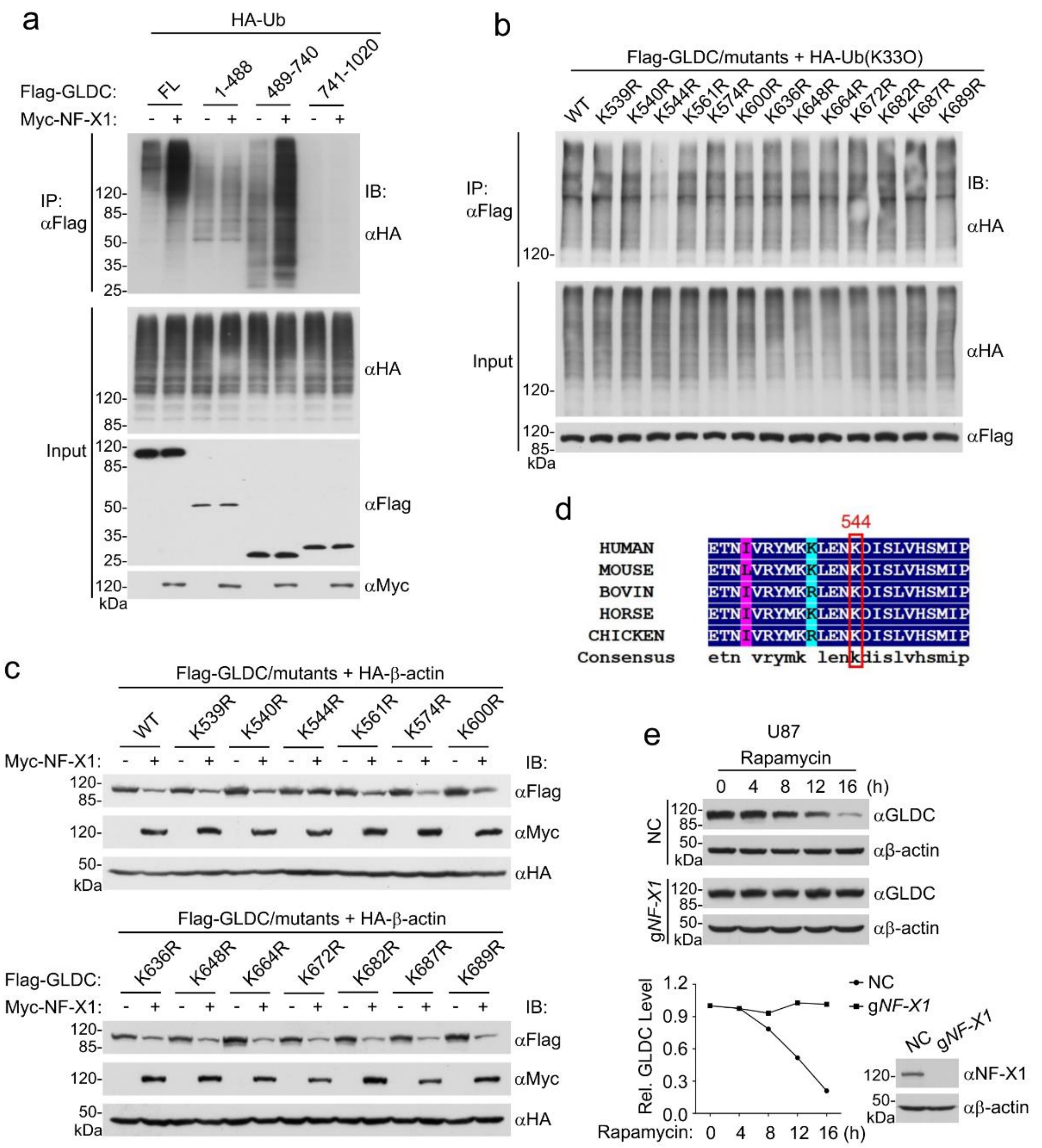

Supplementary Fig. 3. NF-X1 ubiquitinates GLDC at K544. Related to Figure 6.

(a) NF-X1 mediates polyubiquitination of GLDC(489-740). HEK293 cells were transfected with the indicated plasmids for 24 h before co-immunoprecipitation and immunoblotting analysis with the indicated antibodies.

(b) GLDC^{K544R} has reduced K33-linked polyubiquitination. HEK293 cells were transfected with the indicated plasmids for 24 h before co-immunoprecipitation and immunoblotting analysis with the indicated antibodies.

(c) NF-X1 does not down-regulates GLDC^{K544R}. HEK293 cells were transfected with the indicated plasmid for 30 h before immunoblotting analysis with the indicated antibodies.

(d) Sequence alignment of GLDC from the indicated species. The sequences are corresponding to

aa531-554 of hGLDC.

(e) NF-X1-deficiency inhibits Rapamycin-induced degradation of GLDC. Control or NF-X1-deficient U87 cells were un-treated or treated with Rapamycin (50 nM) for the indicated times before immunoblotting analysis. The GLDC band intensities relative to the corresponding β-actin bands were shown in the histograph.

Source data are provided as a source data file.

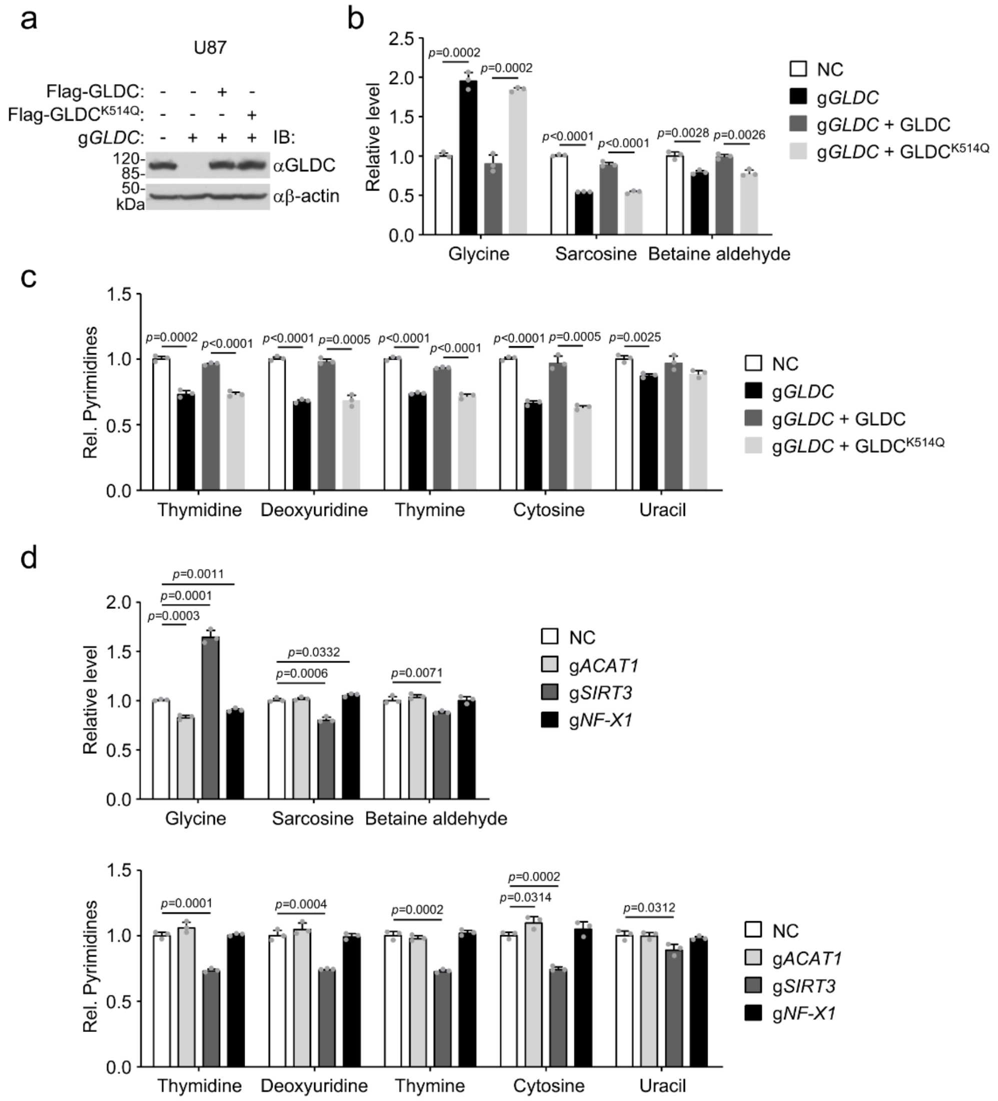

Supplementary Fig. 4. GLDC K514 acetylation inhibits glycine catabolism and pyrimidine synthesis. Related to Figure 8.

(a-c) Reconstitution of GLDC-deficient U87 cells with wild-type GLDC or $GLDC^{K514Q}$. Lysates of the indicated cells were analyzed by immunoblots with the indicated antibodies (a). The control and reconstituted cells were analyzed for levels of glycine and glycine-related metabolites (b) and pyrimidines (c) by LC-HRMS. Graph shows mean ± SEM, n = 3 technical repeats. Data were analyzed using two-way ANOVA with GraphPad Prism 7.

(d) Effects of ACAT1-, SIRT3-, or NF-X1-deficiency on cellular levels of glycine, glycine-related metabolites and pyrimidines. Levels of glycine and its metabolites in U87 cells were measured by LC-HRMS. Graph shows mean ± SEM, n = 3 technical repeats. Data were analyzed using two-way ANOVA with GraphPad Prism 7.

Source data are provided as a source data file.

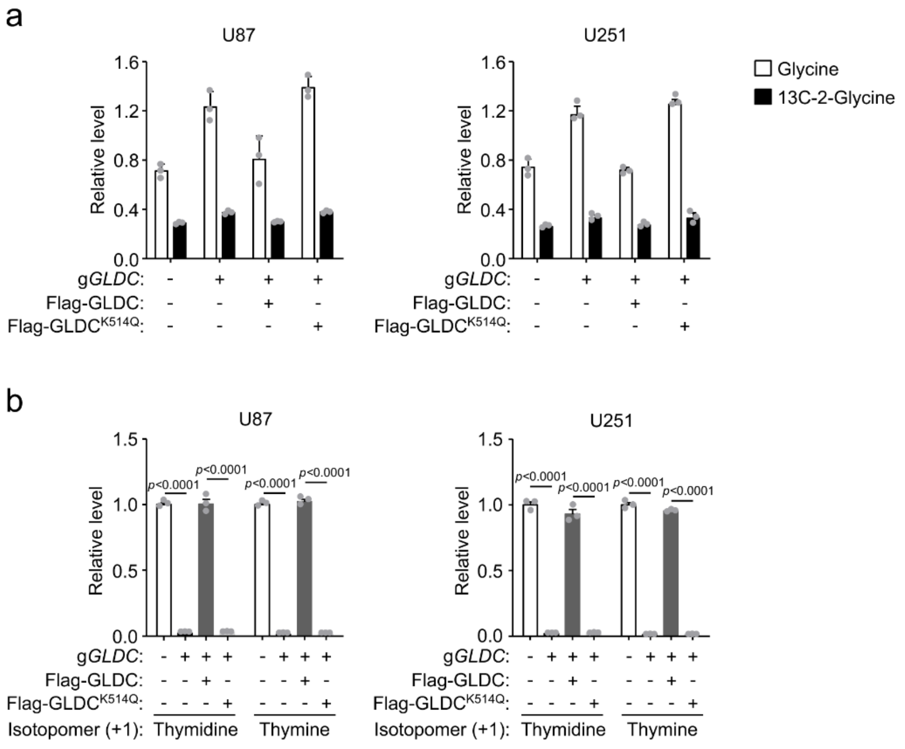

Supplementary Fig. 5. GLDC acetylation impairs *de novo* nucleotide biosynthesis

(a) Abundance of unlabeled or labeled (^{13}C) intracellular glycine in U87 or U251 cells. U87 or U251 cells were cultured in 50% extracellular 13C-2-glycine (0.4 mM glycine + 0.4 mM 13C-2-glycine)-containing medium for 24 h before LC-HRMS analysis. Graph shows mean ± SEM, n = 3 technical repeats.

(b) Effects of GLDC-deficiency or GLDC^{K514Q} reconstitution on cellular levels of 13C-labeled thymidine and thymine. U87 or U251 cells were treated similar as in (a) before LC-HRMS analysis. Graph shows mean ± SEM, n = 3 technical repeats. Data were analyzed using two-way ANOVA with GraphPad Prism 7.

Source data are provided as a source data file.

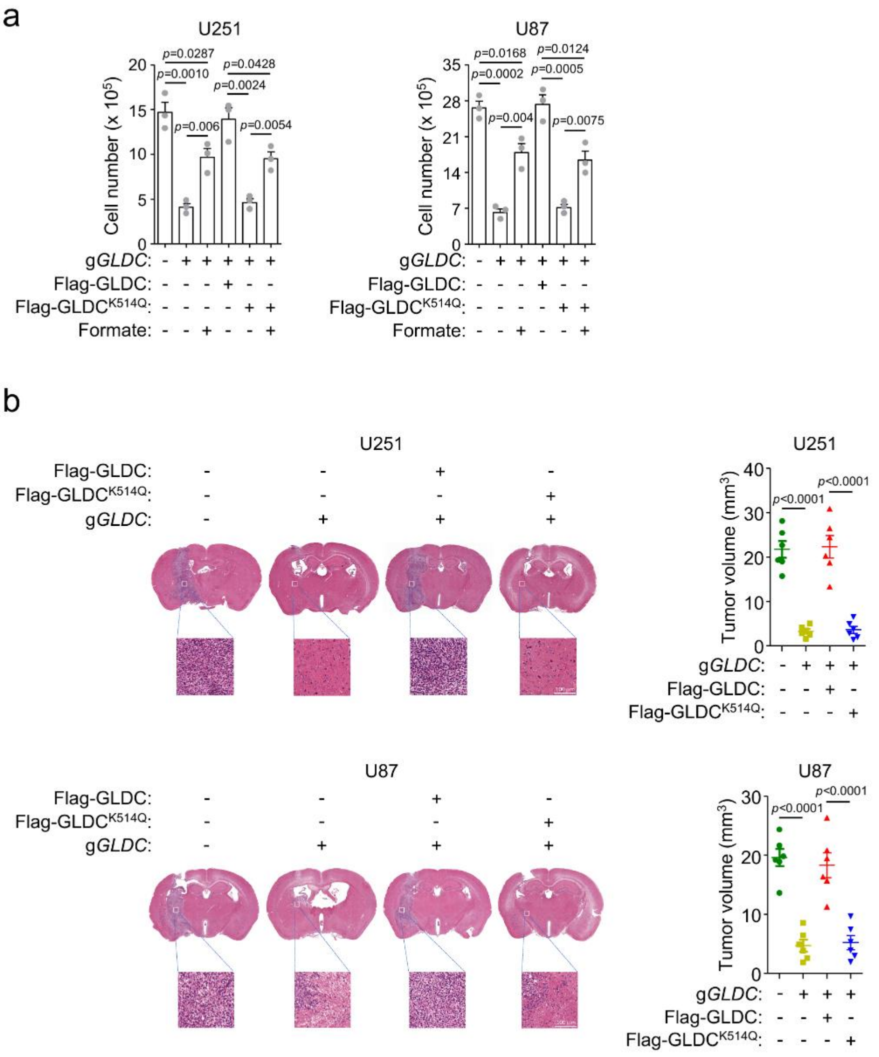

Supplementary Fig. 6. GLDC K514 acetylation suppresses tumorigenesis. Related to Figure 9.

(a) Effects of formate on proliferation of GLDC-deficient or GLDC^{K514Q} cells. Control and GLDC-deficient U251 or U87 cells reconstituted with wild-type GLDC or GLDC^{K514Q} were treated with formate (0.5 mM) for 5 days and then their cell numbers were counted. Graph shows mean ± SEM, n = 3 technical repeats. Data were analyzed using two-way ANOVA with GraphPad Prism 7.

(b) GLDC-deficiency suppresses xenograft tumor growth. Control and GLDC-deficient U251 or U87 cells (5×10^5) reconstituted with wild-type GLDC or GLDC^{K514Q} were intracranially injected into randomized athymic BALB/c nude mice. After 24 (U251) or 18 (U87) days, the mice were euthanized and tumor growth was examined. Hematoxylin and eosin-stained coronal brain sections show representative tumor xenografts (Left panels). Scale bar, 100 μm.

Tumor volumes were measured using length (a) and width (b) and calculated using the equation: $V = ab^2/2$ (Right panels). Graph shows mean ± SEM, n = 6 independent samples. Data were analyzed using two-way ANOVA with GraphPad Prism 7.

Source data are provided as a source data file.

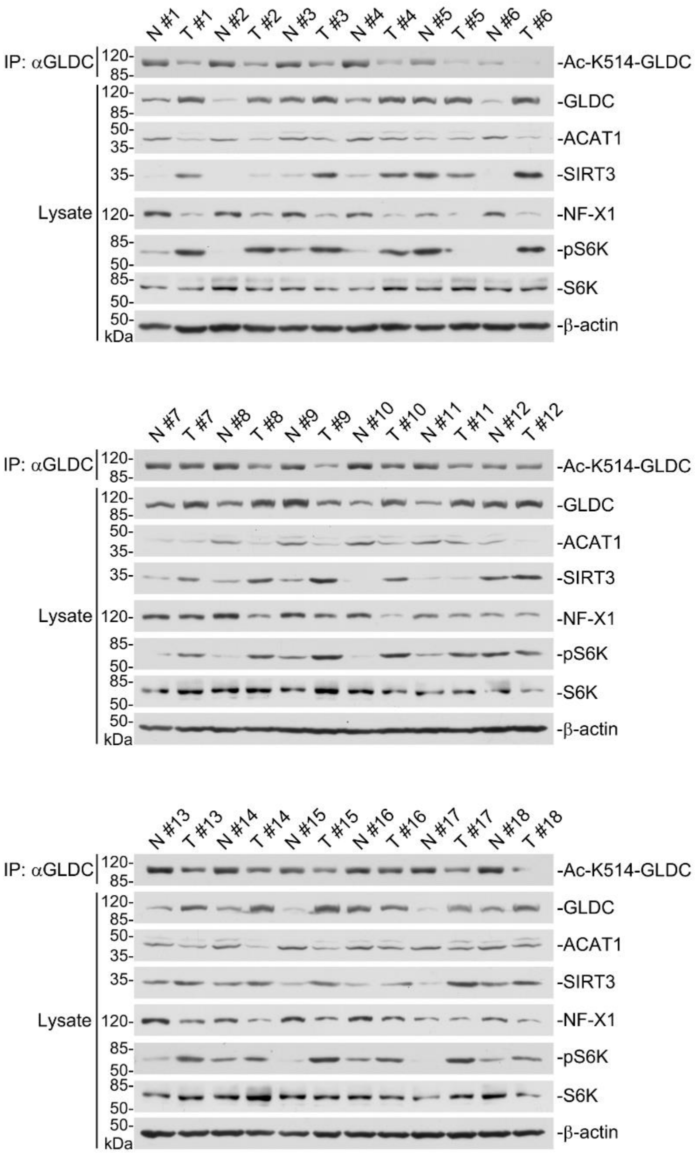

Supplementary Fig. 7. GLDC K514 acetylation is down-regulated in human GBM. Related to Figure 9.

Relative protein levels of the indicated proteins or their modifications. Eighteen non-tumor (N) and 18 GBM (T) samples from patients were subjected to co-immunoprecipitation and immunoblotting analysis with the indicated antibodies. Source data are provided as a source data file.

Supplementary Table 1. A list of primary antibodies used in the study.

Antibody	Supplier	Catalog No.	Appl.[a]	Usage
Mouse anti-Flag M2 antibody clone M2	Sigma-Aldrich	#F3165<br>Lot: SLCC4005	WB/IP	1:2000/1 μg
Mouse anti-HA. 11 Epitope Tag antibody clone 16B12	BioLegend	#901515<br>Lot: B294011	WB/IP	1:2000/1 μg
Mouse anti-Myc-Tag antibody clone 9B11	Cell Signaling Technology	#2276S<br>Lot: 24	WB	1:2000
Mouse anti-β-actin antibody clone AC-74	Sigma-Aldrich	#A2228<br>Lot: 099M47762	WB	1:5000
Mouse anti-β-tubulin antibody	ABclonal	#AC021<br>Lot: 9100010002	WB	1:5000
Mouse anti-SIRT3 antibody clone F-10	Santa Cruz Biotechnology	#sc-365175<br>Lot: F0618	WB	1:500
Mouse anti-NF-X1 antibody clone Y-14	Santa Cruz Biotechnology	#sc-100973<br>Lot: G0617	WB	1:300
Mouse anti-COX IV antibody clone F-8	Santa Cruz Biotechnology	#sc-376731<br>Lot: H2707	WB	1:500
Mouse anti-PGC1α antibody clone D-5	Santa Cruz Biotechnology	#sc-518025<br>Lot: A2319	WB	1:500
Rabbit anti-GLDC antibody	NOVUS	#NBP1-32907<br>Lot: 42326	WB/IP /IF	1:1000/1 μg /1:400
Rabbit anti-ACAT1 antibody	Cell Signaling Technology	#44276<br>Lot: 1	WB	1:1000
Rabbit anti-RPTOR antibody clone E6O3A	Cell Signaling Technology	#48648<br>Lot: 12	WB	1:1000
Rabbit anti-pS6K (Thr389) antibody clone 108D2	Cell Signaling Technology	#9234<br>Lot: 21	WB	1:1000
Rabbit anti-p4EBP1 (Thr37/46) antibody clone 236B4	Cell Signaling Technology	#2855<br>Lot: 23	WB	1:1000
Rabbit anti-S6K antibody	Proteintech	#14485-1-AP<br>Lot: 00022879	WB	1:8000
Rabbit anti-4EBP1 antibody clone 2C3F3	Proteintech	#60246-1-Ig<br>Lot: 10004043	WB	1:3500
Rabbit anti-K33-linkage specific polyubiquitination antibody	ABclonal	#A18199<br>Lot: 3561211108	WB	1:500
Rabbit anti-Acetyl antibody	Abcam	#ab21623<br>Lot: GR3203593-9	WB	1:2000
Mouse anti-p-Tyr antibody clone P-Tyr-100	Cell Signaling Technology	#9411<br>Lot: 27	WB	1:1000
Rabbit anti-p-Ser/Thr antibody	Abcam	#ab17464<br>Lot: GR3346404-1	WB	1:1000

Supplementary Table 2. The clinicopathologic characteristics in patients.

Patient characteristics	Non-glioma	Glioma
**Age (years)**		
≤45	8	13
>45	10	5
**Sex**		
Male	12	11
Female	6	7
**Diagnosis**	Brain trauma	Glioma
**Location of tissue**		
Frontal lobe	2	4
Parietal lobe	7	5
Temporal lobe	9	8
Occipital lobe	0	1

Supplementary Table 3. A list of gRNA sequences.

Human *GLDC*	5’-CAGATCTGGAGATCGTATAT-3’
Human *ACAT1*	5’-TACTCAGCCCTCTGCGACCA-3’
Human *SIRT3*	5’- GTAGTTGAACGGGTCGAGGC-3’
Human *PGC1α*	5’-GGCGTGGGACATGTGCAACC-3’
Human *RPTOR*	5’-ATGCAGTTTTTCATCGACGG-3’
Human *NF-X1*	5’-GGCCACCGACATACAAACGC-3’

Supplementary Table 4. A list of qPCR sequences.

GAPDH	GTCTCCTCTGACTTCAACAGCG
	ACCACCCTGTTGCTGTAGCCAA
GLDC	GCTTGGTGAGAATGATGCCTGG
	CAGATGTTGCTGGTAGCCTTGTC
SIRT3	CCCTGGAAACTACAAGCCCAAC
	GCAGAGGCAAAGGTTCCATGAG
