# Supplementary material for: mTORC1 activity regulates post-translational modifications of glycine decarboxylase to modulate glycine metabolism and tumorigenesis
Source: Nat Commun. 2021 Jul 9;12:4227. doi: 10.1038/s41467-021-24321-3 (PMC8270999; doi:10.1038/s41467-021-24321-3)
Supplement: Supplementary file 2 — Description of Additional Supplementary Files [file 41467_2021_24321_MOESM2_ESM.pdf]

Description of Additional Supplementary Files

Supplementary Data 1: Hits of GLDC-associated proteins in mass spectrometry analysis
