# Supplementary material for: mTORC1 activity regulates post-translational modifications of glycine decarboxylase to modulate glycine metabolism and tumorigenesis
Source: Nat Commun. 2021 Jul 9;12:4227. doi: 10.1038/s41467-021-24321-3 (PMC8270999; doi:10.1038/s41467-021-24321-3)
Supplement: Supplementary file 4 — Reporting summary [file 41467_2021_24321_MOESM4_ESM.pdf]

nature research

Corresponding author(s): Shu Li

Last updated by author(s): May 20, 2021

Reporting Summary

Nature Research wishes to improve the reproducibility of the work that we publish. This form provides structure for consistency and transparency in reporting. For further information on Nature Research policies, see our Editorial Policies and the Editorial Policy Checklist.

Statistics

For all statistical analyses, confirm that the following items are present in the figure legend, table legend, main text, or Methods section.

n/a	Confirmed	
☐	☒	The exact sample size (n) for each experimental group/condition, given as a discrete number and unit of measurement
☐	☒	A statement on whether measurements were taken from distinct samples or whether the same sample was measured repeatedly
☐	☒	The statistical test(s) used AND whether they are one- or two-sided<br>*Only common tests should be described solely by name; describe more complex techniques in the Methods section.*
☒	☐	A description of all covariates tested
☒	☐	A description of any assumptions or corrections, such as tests of normality and adjustment for multiple comparisons
☐	☒	A full description of the statistical parameters including central tendency (e.g. means) or other basic estimates (e.g. regression coefficient) AND variation (e.g. standard deviation) or associated estimates of uncertainty (e.g. confidence intervals)
☒	☐	For null hypothesis testing, the test statistic (e.g. F, t, r) with confidence intervals, effect sizes, degrees of freedom and P value noted<br>*Give P values as exact values whenever suitable.*
☒	☐	For Bayesian analysis, information on the choice of priors and Markov chain Monte Carlo settings
☒	☐	For hierarchical and complex designs, identification of the appropriate level for tests and full reporting of outcomes
☒	☐	Estimates of effect sizes (e.g. Cohen's d, Pearson's r), indicating how they were calculated

Our web collection on statistics for biologists contains articles on many of the points above.

Software and code

Policy information about availability of computer code

Data collection	The qRT-PCR data: Bio-Rad CFX96 (Version 3.1)<br>The Liquid chromatography-high resolution mass spectrometry (LC-HRMS) data: Thermo LTQ Obitrap Elite
Data analysis	Statistical analysis: GraphPad Prism (ver. 7)<br>qRT-PCR analysis: Bio-Rad CFX Manager (Version 3.1)<br>Western Blot quantitation: ImageJ (Version1.8.0, developed by NIH)

For manuscripts utilizing custom algorithms or software that are central to the research but not yet described in published literature, software must be made available to editors and reviewers. We strongly encourage code deposition in a community repository (e.g. GitHub). See the Nature Research guidelines for submitting code & software for further information.

Data

Policy information about availability of data

All manuscripts must include a data availability statement. This statement should provide the following information, where applicable:
- Accession codes, unique identifiers, or web links for publicly available datasets
- A list of figures that have associated raw data
- A description of any restrictions on data availability

All the data supporting the findings of this study are available within the article and its supplementary information files, or can be obtained from the corresponding author upon reasonable request. A reporting summary for this article is available as a Supplementary Information file. Source data are provided with this paper.

Field-specific reporting

Please select the one below that is the best fit for your research. If you are not sure, read the appropriate sections before making your selection.

- [x] Life sciences
- [ ] Behavioural & social sciences
- [ ] Ecological, evolutionary & environmental sciences

For a reference copy of the document with all sections, see nature.com/documents/nr-reporting-summary-flat.pdf

Life sciences study design

All studies must disclose on these points even when the disclosure is negative.

Sample size	No statistical methods were used to predetermine sample size. The sample sizes were determined based on the previous studies, and the sizes are large enough to ensure the credibility of results.
Data exclusions	No data were excluded throughout the studies.
Replication	The replication numbers were described in the corresponding figure legends.
Randomization	For in vitro experiments, cells were randomly allocated into control and experimental groups. For in vivo experiments, age and sex-matched mice were randomized into all experimental groups. Samples of noncancerous individuals and cancer patients were collected and analyzed identically regardless of other clinical history or external criteria.
Blinding	Data collection of mouse tumor experiments were performed in a double blinding manner, the investigators were blinded to group allocation during 'data analysis' of mouse tumor experiments. Cellular and biochemical experiments were not performed in a blinding manner because the same investigator was doing group allocation during data collection and/or analysis. For other experiments, investigators were not blinded to the identity of samples to ensure appropriate data collection and because experimental results are quantitative in nature, not readily subject to investigator bias. To ensure consistent experimental conditions, all control and experimental samples were processed in parallel.

Reporting for specific materials, systems and methods

We require information from authors about some types of materials, experimental systems and methods used in many studies. Here, indicate whether each material, system or method listed is relevant to your study. If you are not sure if a list item applies to your research, read the appropriate section before selecting a response.

Materials & experimental systems

n/a	Involved in the study
☐	☒ Antibodies
☐	☒ Eukaryotic cell lines
☒	☐ Palaeontology and archaeology
☐	☒ Animals and other organisms
☐	☒ Human research participants
☒	☐ Clinical data
☒	☐ Dual use research of concern

Methods

n/a	Involved in the study
☒	☐ ChIP-seq
☒	☐ Flow cytometry
☒	☐ MRI-based neuroimaging

Antibodies

Antibodies used	Antibodies used in immunoblots, immunoprecipitation and immunofluorescence experiments were listed in Supplementary Table 2.
Validation	The WB antibody for human Ac-K514-GLDC was validated in GLDC-deficient U251 cells (Fig. 1F). All other antibodies in the study were bought commercially. We provided the validation of the antibodies for the species and application in our experiments, as well as all commercially antibodies have been validated either from prior reports and studies or validated by the manufacturer as stated on the websites from the catalog numbers listed above or published references on the manufacturers' websites. Manufacturers state the antibodies have been validated for intended uses. Manufacturer citations are listed in manufacturer websites for each specific antibody.

Eukaryotic cell lines

Policy information about cell lines

Cell line source(s)	U87, U251 cells and HEK293 cells were obtained from ATCC.
Authentication	None of the cell lines have been authenticated.

Mycoplasma contamination	All cell lines were tested negative for mycoplasma contamination.
Commonly misidentified lines (See ICLAC register)	No commonly mis-identified cell lines were used.

Animals and other organisms

Policy information about studies involving animals; ARRIVE guidelines recommended for reporting animal research

Laboratory animals	BALB/c nude mice were purchased from GemPharmatech (Jiangsu, China). All mice were housed with 5 mice per cage on a 12 h light/dark cycle in a temperature-controlled room (23-25°C) and relative humidity of 40-70% with free access to water and food. Eight to ten week-old and age-matched male mice were used in all experiments.
Wild animals	The study did not involve wild animals.
Field-collected samples	The study did not involve samples collected from the field.
Ethics oversight	All animal use and experimental protocols were approved and carried out in compliance with the Institutional Animal Care and Use Committee (IACUC) guidelines and the Animal Care and Ethics Committee of Wuhan University Medical Research Institute.

Note that full information on the approval of the study protocol must also be provided in the manuscript.

Human research participants

Policy information about studies involving human research participants

Population characteristics	Human glioma and non-glioma tissues were collected from the Department of Neurosurgery, Renmin Hospital of Wuhan University, Wuhan, China. Non-glioma tissues were collected during surgery of severe traumatic brain injury after informed consent from the patients who needed post-trauma surgery. The clinical glioma specimens were examined and diagnosed by pathologists at Renmin Hospital of Wuhan University. The detailed clinicopathologic characteristics of patients is presented in Supplementary Table 2.
Recruitment	The clinical samples were collected at the Department of Neurosurgery, Renmin Hospital of Wuhan University, Wuhan, China. Participants were recruited as a part of traumatic brain injury patients or glioma patients.
Ethics oversight	Tissue procurement and use in this study were performed with written patient informed consents and approved by the Institutional Ethics Committee of Renmin Hospital of Wuhan University

Note that full information on the approval of the study protocol must also be provided in the manuscript.
